# Biallelic *NDUFA13* variants lead to a neurodevelopmental phenotype with gradual neurological impairment

**DOI:** 10.1093/braincomms/fcae453

**Published:** 2024-12-17

**Authors:** Rauan Kaiyrzhanov, Kyle Thompson, Stephanie Efthymiou, Askhat Mukushev, Akbota Zharylkassyn, Chitra Prasad, Ehsan Ghayoor Karimiani, Javeria Raza Alvi, Dmitriy Niyazov, Ahmad Alahmad, Meisam Babaei, Homa Tajsharghi, Buthaina Albash, Ahmad Alaqeel, Majida Charif, Narges Hashemi, Morteza Heidari, Seyed Mehdi Kalantar, Guy Lenaers, Mohammad Yahya Vahidi Mehrjardi, Varunvenkat M Srinivasan, Vykuntaraju K Gowda, Seyed Hamidreza Mirabutalebi, Deanna Alexis Carere, Mojtaba Movahedinia, David Murphy, Robert McFarland, Mohamed S Abdel-Hamid, Rasha M Elhossini, Shahryar Alavi, Melanie Napier, Amaya Belanger-Quintana, Asuri N Prasad, Jessica Jakobczyk, Agathe Roubertie, Tony Rupar, Tipu Sultan, Mehran Beiraghi Toosi, Leonid Sazanov, Mariasavina Severino, Henry Houlden, Robert W Taylor, Reza Maroofian

**Affiliations:** Department of Neuromuscular Diseases, Queen Square Institute of Neurology, University College London, London WC1N 3BG, UK; Department of Neurology, South Kazakhstan Medical Academy, Shymkent 160019, Kazakhstan; Mitochondrial Research Group, Translational and Clinical Research Institute, Faculty of Medical Sciences, Newcastle University, Newcastle upon Tyne NE2 4HH, UK; Department of Neuromuscular Diseases, Queen Square Institute of Neurology, University College London, London WC1N 3BG, UK; Department of Neurology, Beth Israel Deaconess Medical Center, Harvard Medical School, Boston, MA 02215-5400, USA; The Institute of Childhood Neurology, Almaty 050000, Kazakhstan; Division of Genetics and Metabolics, Department of Pediatrics, London Health Sciences, London, ON, Canada N6A 5W9; Department of Neuromuscular Diseases, Queen Square Institute of Neurology, University College London, London WC1N 3BG, UK; Molecular and Clinical Sciences Institute, St. George’s University of London, London SW17 0RE, UK; Department of Pediatric Neurology, Children's Hospital and Institute of Child Health, Lahore 54000, Pakistan; Department of Pediatrics, Duke University School of Medicine, Durham, NC 27710, USA; Molecular Genetics Laboratory, Kuwait Medical Genetics Center, Ministry of Health, Sulaibikhat 80901, Kuwait; Department of Pediatrics, North Khorasan University of Medical Sciences, Bojnurd 9413813965, Iran; School of Health Sciences, Division of Biomedicine, University of Skovde, Skovde 541 28, Sweden; Kuwait Medical Genetics Centre, Al-Sabah Medical Area, Kuwait City 80901, Kuwait; Kuwait Medical Genetics Centre, Al-Sabah Medical Area, Kuwait City 80901, Kuwait; Genetics Unit, Medical Sciences Research Laboratory, Faculty of Medicine and Pharmacy, University Mohammed Premier, Oujda 60000, Morocco; BRO Biobank, Faculty of Medicine and Pharmacy, University Mohammed Premier, Oujda 60000, Morocco; Genetic and Immuno-Cell Therapy Team, Mohammed First University, Oujda 60000, Morocco; Department of Pediatrics, School of Medicine, Mashhad University of Medical Sciences, Mashhad 91778 99191, Iran; Myelin Disorders Clinic, Department of Pediatric Neurology, Children’s Medical Center, Pediatrics Center of Excellence, Tehran University of Medical Sciences, Tehran 14197 33151, Iran; Abortion Research Centre, Yazd Reproductive Sciences Institute, Shahid Sadoughi University of Medical Sciences, Yazd 8916188635, Iran; Angers University, MitoLab Team, MitoVasc Unit, CNRS UMR6015, INSERM U1083, SFR ICAT, Angers 49035, France; Department of Neurology, University Hospital of Angers, Angers 49035, France; Diabetes Research Center, Shahid Sadoughi University of Medical Sciences, Yazd 8916188635, Iran; Department of Pediatric Neurology, Indira Gandhi Institute of Child Health, Bangalore 560 029, India; Department of Pediatric Neurology, Indira Gandhi Institute of Child Health, Bangalore 560 029, India; Abortion Research Centre, Yazd Reproductive Sciences Institute, Shahid Sadoughi University of Medical Sciences, Yazd 8916188635, Iran; GeneDx Inc., Gaithersburg, MD 20877, USA; Children Growth Disorder Research Center, Shahid Sadoughi University of Medical Sciences, Yazd 8916188635, Iran; Department of Clinical and Movement Neurosciences, UCL Queen Square Institute of Neurology, London WC1N 3BG, UK; Mitochondrial Research Group, Translational and Clinical Research Institute, Faculty of Medical Sciences, Newcastle University, Newcastle upon Tyne NE2 4HH, UK; NHS Highly Specialised Service for Rare Mitochondrial Disorders, Newcastle upon Tyne Hospitals NHS Foundation Trust, Newcastle upon Tyne NE1 4LP, UK; Medical Molecular Genetics Department, Human Genetics and Genome Research Institute, National Research Centre, Cairo 12622, Egypt; Clinical Genetics Department, Human Genetics and Genome Research Institute, National Research Centre, Cairo 12622, Egypt; Department of Neuromuscular Diseases, Queen Square Institute of Neurology, University College London, London WC1N 3BG, UK; GeneDx Inc., Gaithersburg, MD 20877, USA; Servicio de Pediatría, Enfermedades Metabólicas Hereditarias, Hospital Universitario Ramón y Cajal, Madrid 28034, Spain; Division of Pediatric Neurology, Department of Pediatrics, Western University, London, ON, Canada N6A 5W9; Division of Genetics and Metabolics, Department of Pediatrics, London Health Sciences, London, ON, Canada N6A 5W9; Department of Neuropaediatrics, Gui de Chauliac Hospital, Montpellier University Hospital, Institut des Neurosciences, INSERM U 1298, Montpellier 34091, France; Department of Pediatrics, University of Western Ontario, London, ON, Canada N6A5W9; Departments of Biochemistry, Pathology and Laboratory Medicine, University of Western Ontario, London, ON, Canada N6A5W9; Department of Pediatric Neurology, Children's Hospital and Institute of Child Health, Lahore 54000, Pakistan; Department of Pediatrics, School of Medicine, Mashhad University of Medical Sciences, Mashhad 91778 99191, Iran; Neuroscience Research Center, Mashhad University of Medical Sciences, Mashhad 91778 99191, Iran; Institute of Science and Technology Austria, Klosterneuburg A-3400, Austria; Neuroradiology Unit, IRCCS Istituto Giannina Gaslini, Genoa 16147, Italy; Department of Neuromuscular Diseases, Queen Square Institute of Neurology, University College London, London WC1N 3BG, UK; Mitochondrial Research Group, Translational and Clinical Research Institute, Faculty of Medical Sciences, Newcastle University, Newcastle upon Tyne NE2 4HH, UK; NHS Highly Specialised Service for Rare Mitochondrial Disorders, Newcastle upon Tyne Hospitals NHS Foundation Trust, Newcastle upon Tyne NE1 4LP, UK; Department of Neuromuscular Diseases, Queen Square Institute of Neurology, University College London, London WC1N 3BG, UK

**Keywords:** complex I deficiency, mitochondrial disorders, NDUFA13, neurodevelopmental disorder, Leigh syndrome

## Abstract

Biallelic variants in NADH (nicotinamide adenine dinucleotide (NAD) + hydrogen (H))-ubiquinone oxidoreductase 1 alpha subcomplex 13 have been linked to mitochondrial complex I deficiency, nuclear type 28, based on three affected individuals from two families. With only two families reported, the clinical and molecular spectrum of NADH-ubiquinone oxidoreductase 1 alpha subcomplex 13*–*related diseases remains unclear. We report 10 additional affected individuals from nine independent families, identifying four missense variants (including recurrent c.170G > A) and three ultra-rare or novel predicted loss-of-function biallelic variants. Updated clinical–radiological data from previously reported families and a literature review compiling clinical features of all reported patients with isolated complex I deficiency caused by 43 genes encoding complex I subunits and assembly factors are also provided. Our cohort (mean age 7.8 ± 5.4 years; range 2.5–18) predominantly presented a moderate-to-severe neurodevelopmental syndrome with oculomotor abnormalities (84%), spasticity/hypertonia (83%), hypotonia (69%), cerebellar ataxia (66%), movement disorders (58%) and epilepsy (46%). Neuroimaging revealed bilateral symmetric T2 hyperintense substantia nigra lesions (91.6%) and optic nerve atrophy (66.6%). Protein modeling suggests missense variants destabilize a critical junction between the hydrophilic and membrane arms of complex I. Fibroblasts from two patients showed reduced complex I activity and compensatory complex IV activity increase. This study characterizes NADH-ubiquinone oxidoreductase 1 alpha subcomplex 13*–*related disease in 13 individuals, highlighting genotype–phenotype correlations.

## Teaser

Kaiyrzhanov *et al*. provide a cumulative phenotype characterization of NADH-ubiquinone oxidoreductase 1 alpha subcomplex 13 (*NDUFA13*)-related disease describing 13 patients, 10 of whom are previously unpublished. The study reveals the core phenotype of the *NDUFA13*-related disease as well as its clinical spectrum. This study aids in understanding *NDUFA13*-related complex I deficiency.

## Graphical Abstract

**Graphical Abstract:**
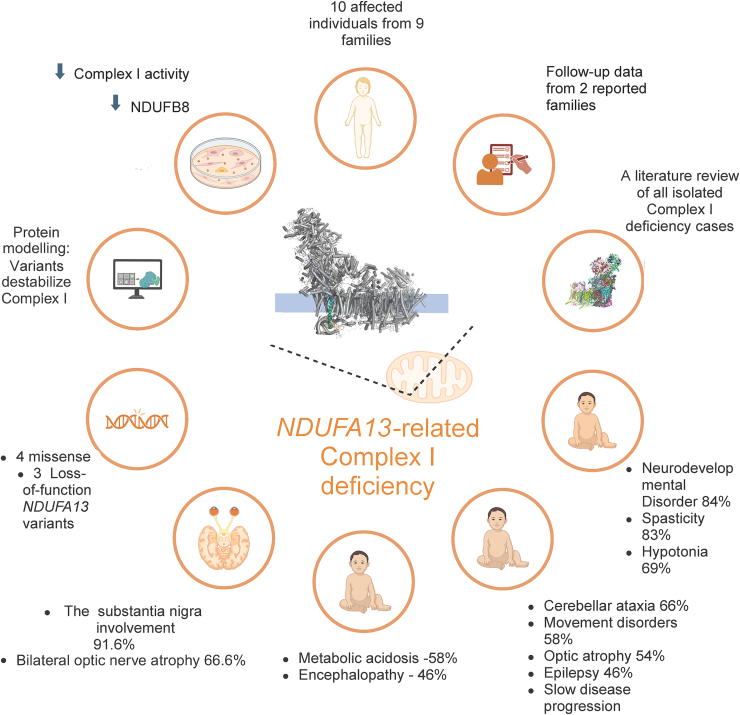

## Introduction

NADH (nicotinamide adenine dinucleotide (NAD) + hydrogen (H))-ubiquinone oxidoreductase 1 alpha subcomplex 13 (*NDUFA13*) (MIM*609435) forms one of the 44 subunits of the mitochondrial respiratory complex I (CI).^1^ *NDUFA13* has a broad range of functions, the most important of which is maintaining the mitochondrial membrane potential by controlling CI assembly and activity.^2^ *NDUFA13* is also known as gene associated with retinoid-interferon-induced mortality 19 and is reported to control interferon-beta and retinoic acid–induced cell death and expresses antioncogenic function negatively regulating signal transducer and transcription activator).^3^ The latter mediates cellular responses to cytokines and various growth factors.^3-5^ *NDUFA13*-null mice result in embryonic lethality, whereas blastocysts with homozygous *NDUFA13* deletion show absent mitochondrial CI.^6^

CI deficiency is the most frequent early-onset mitochondrial oxidative phosphorylation (OXPHOS) disorder accounting for up to 30% of cases.^7^ Biallelic variants in *NDUFA13* have been associated with mitochondrial CI deficiency, nuclear type 28 in three affected individuals from two families.^8,9^ Currently, with only two families reported, the clinical and molecular spectrum of *NDUFA13*-related CI deficiency remains poorly characterized; hence, here, we report on further 10 affected individuals from 9 independent families with four rare missense and three predicted loss-of-function (LOF) biallelic *NDUFA13* variants and provide follow-up clinical-radiological details from the previously reported families. Our study provides a cumulative phenotypic characterization of *NDUFA13*-related disease.

## Materials and methods

### Patient identification, genetic and clinical investigation

Using the GeneMatcher platform^10^ and extensive international data sharing, nine families reported here were identified. A uniform clinical proforma was distributed to obtain clinical details from new and previously reported families. Brain MRI was available in 12 individuals from 10 families and was reviewed by an experienced paediatric neuroradiologist (M.S.). Parents and legal guardians of all affected individuals gave their consent for the publication of clinical and genetic information according to the Declaration of Helsinki, and the study was approved by the respective local Ethics Committees.

Proband-only or trio exome sequencings were performed on DNA extracted from blood-derived leukocytes, followed by data analysis and variant filtration, and Sanger segregation analysis in 9 families in 6 different centres following the protocols described previously (Supplemental Table 1). Haplotype analysis was done using our ExHap tool. Briefly, VCF files of patients harbouring shared pathogenic variant were merged along with control samples. Using R, we plotted a colour banding plot to visualize genotype pattern of flanking regions of the variant site to infer homozygosity haplotypes.

### The assessment of OXPHOS protein expression by western blotting and the evaluation of mitochondrial respiratory chain activities in fibroblasts

Skin biopsies were obtained from two families (Individuals 7 and 8), establishing explant cultures to grow primary fibroblast cell lines. Cells were prepared, alongside two paediatric control lines, for whole-cell lysates and subsequent SDS-PAGE/western blot analysis as described previously.^11^ The assessment of mitochondrial respiratory chain enzyme activities was made in mitochondrial fractions as described previously.^12^ See Supplementary methods for further details.

### Protein modelling

The variants were considered in the context of cryo-EM human CI structure from PDB 5XTD.^13^ The conservation of the structure was evaluated by alignment to the ovine CI structure (PDB 6ZKC). The interactions of mutated residues were evaluated by considering polar interactions within the program PYMOL.

### Literature review

We compiled a list of genes associated with CI deficiency using the Online Mendelian Inheritance in Man (OMIM) database. Multiple electronic databases including PubMed, Web of Science, and Google Scholar were screened with the search terms including ‘Complex I deficiency’, ‘NADH oxidoreductase’, ‘mitochondrial disease’ and the names of the target genes. All identified case reports and case series with original patient data published in English and providing phenotypic presentation were considered. Articles not related to CI deficiency and reviews, editorials and opinion pieces without original patient data were excluded. For each included study, we extracted relevant clinical details using a standardized data collection form. Extracted data were tabulated and analysed to identify common clinical patterns, genotype–phenotype correlations and variations in disease presentation among CI deficiency-associated genes. Based on the frequency of reported symptoms, we extracted core phenotypes for each CI deficiency-associated gene.

No statistical analysis was performed in this study.

## Results

### Clinical findings

**Figure 1:**
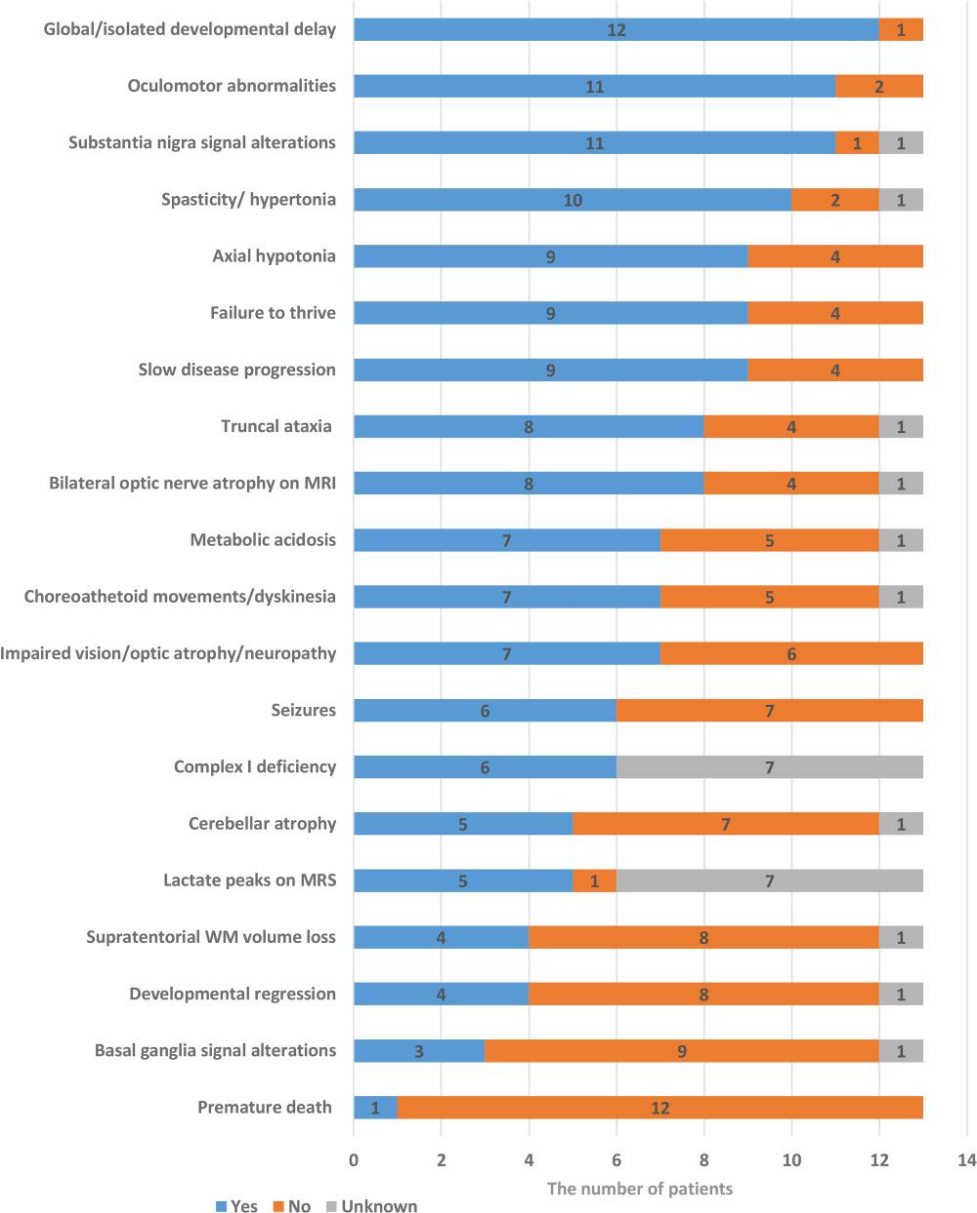
**Clinical–radiological features**. Clinical features of the affected individuals with biallelic *NDUFA13* variants. The bar chart illustrates the most common clinical symptoms and signs associated with *NDUFA13*-related disease, presented in descending order of frequency. The *x*-axis represents the total number of patients (*N* = 13), while the *y*-axis lists the symptoms and signs. The blue segments indicate the number of patients exhibiting these symptoms/signs, the orange segments show the number of patients without them and the grey segments represent patients for whom the presence or absence of these symptoms/signs is unconfirmed. MRS, magnetic resonance spectroscopy.

The main phenotypic features of the present cohort and the previously reported individuals affected by *NDUFA13-*related disease are summarized in Fig. 1, Supplemental Tables 1 and 2. Detailed clinical history is provided in the supplemental case reports. Video recordings are available for families 1, 2, and 7 (Videos 1–3). The further clinical description will focus on 13 affected individuals from 11 unrelated families including 9 families found in the present study and two previously reported families with their follow-up details.^8,9^ There were 7 females and 6 males, 12 of whom are currently alive with a mean age of 7.8 ± 5.4 years (range 2.5–18). The disease was mostly infantile-onset (≤12 months) except for two affected individuals manifesting after the age of 18 months old (Families 2 and 8). The onset of the disease was more subtle and progressive, rather than being associated with decompensation during a somatic illness, vaccination or fever. In more than half of the individuals (8/13, 61%), the disease had slow rates of progression, whereas others displayed moderate (3/13, 23%) and rapid (2/13, 16%) rates of progression. The type of progression was determined subjectively by clinicians based on the degree of acquisition of milestones, regression, accumulation of symptoms and their severity and functional capacity. Failure to thrive was reported in most of the cohort (9/13, 69%). A history of metabolic acidosis was present in 7/12 (58%) individuals.

Developmental delay was severe-to-profound in 7/13 (54%), moderate in 5/13 (38%) and mild in 1/13 (8%) affected individuals. While eight affected individuals presented with global developmental delay, four affected individuals had only isolated motor delay. Half of the affected individuals (7/13, 53%) had never attained independent walking, two affected individuals walked independently by 2 years old but then gradually lost independent ambulation, and three individuals were able to walk albeit with an ataxic gait. The speech was mostly limited to sounds only and babbling (7/13, 54%) except for the other six individuals who were able to talk.

The median age of the cohort at the most recent follow-up was 4.7 years (range, 2.3–17) where the affected individuals displayed moderate-to-severe intellectual disability (8/13, 61%), acquired microcephaly (4/13, 31%) and encephalopathy (6/13, 46%). Reduced visual acuity with bilateral optic atrophy or neuropathy (7/13, 54%) and oculomotor abnormalities (11/13, 84%) including nystagmus, slow saccades, ptosis and esotropia were among the frequent ocular signs. Cataracts were documented to be absent in 8/13 patients, and its presence is unknown in five. Neuromuscular phenotype included dysarthria (8/13, 61%), axial (9/13, 69%) and peripheral (6/12, 50%) hypotonia (in the infantile period) and spasticity/hypertonia (10/12, 83%). Movement disorders including choreoathetoid movements of the limbs and face (7/12, 58%), dyskinesia (7/12, 58%), bradykinesia (5/11, 45%) and truncal ataxia (8/12, 66%) were frequently displayed in the cohort. Hyperkinetic movement disorders tended to subside over time in 5/6 (83%) affected individuals. Generalized tonic-clonic or myoclonic seizures were reported in 6/13 (46%) individuals with seizure manifestation from the age of 3 months to 9 years. No pharmacoresistant seizures were reported.

Biochemical tests from serum and urine were available from 8 patients, which revealed increased serum lactate (7/8 patients with a mean serum lactate level of 4.1 ± 07 mmol/L; range, 3.2–5.0; normal levels <2 mmol/L) and increased serum or urine alanine (3/8). Lactate levels in the cerebrospinal fluid were available from 6/13 patients and were increased in two individuals (2.8 and 3 mmol/l, normal range −1.0 to 2.0 mmol/L). Only six individuals in the cohort underwent respiratory chain enzyme testing, either in muscle tissue or skin fibroblasts, which confirmed isolated CI deficiency in every case.

**Figure 2:**
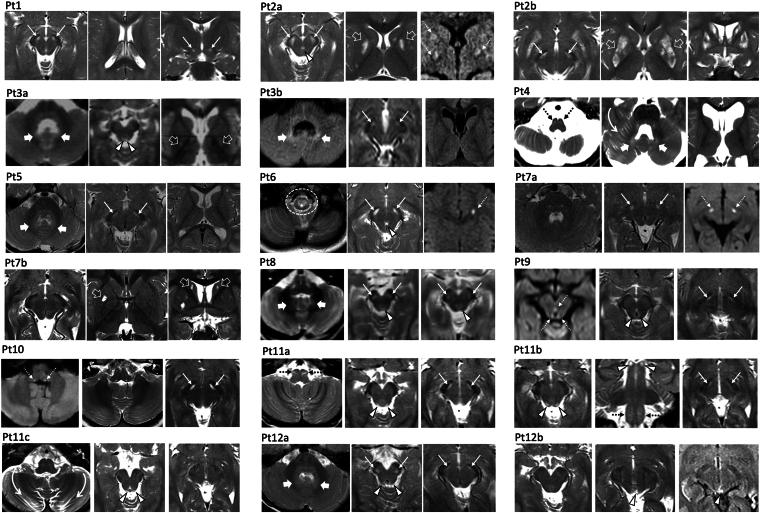
**Neuroimaging features of *NDUFA13*-related disease**. Most of the individuals present symmetric T2-hyperintense lesions of the substantia nigra (thin white arrows). These lesions are less evident on follow-up scans, as seen in Patient 2b, Patient 7b, and Patient 11b and c and Patient 12b). Periaqueductal and central midbrain signal alterations are visible in individuals Patient 3, Patient 6, Patient 9, Patient 11 and Patient 12 (white arrowheads). In three patients (Patient 2, Patient 3 and Patient 7), there are additional basal ganglia signal alterations (empty white arrows), which increase over time with associated atrophy in individual Patient 2, as depicted in Patient 2b. Faint T2 hyperintensities of the dentate nuclei are noticed in individuals Patient 3, Patient 4, Patient 5, Patient 8 and Patient 12 (thick white arrows). Subtle inferior olivary nuclei T2 hyperintensity is visible in individuals Patient 4 and Patient 11 (dashed black arrows). In one individual (Patient 6), there are T2 signal alterations at the level of the cervical spinal cord (dashed oval). Small foci of restricted diffusion (dashed white arrows) are noted in the right basal ganglia and bilateral substantia nigra (Patient 2a), left periventricular frontal white matter (Patient 6), bilateral substantia nigra (Patient 7a), periaqueductal grey matter (Patient 9) and posterior medullary white matter tracts (Patient 10). Note: mild cerebellar atrophy mainly involving the cerebellar hemispheres in Patients 4 and 11 (curved arrows in Patient 4 and Patient 11c). Pt, patient.

Nineteen brain MRI studies were available for review in 12 individuals from 10 families (mean age at first MRI, 2.4 years; range, 9 months to 7 years). Frequent neuroimaging features (Fig. 2) included bilateral symmetric T2 hyperintense lesions in the substantia nigra (11/12, 91.6%) and bilateral optic nerve atrophy (8/12, 66.6%). Faint signal alterations in the cerebellar dentate nuclei were noted in 7/12 cases (58.3%), while periaqueductal grey matter focal lesions were present in 6/12 affected individuals (50%). Mild cerebellar atrophy involving the lateral or superior portions of the cerebellar hemispheres was depicted in 5/12 individuals (41.6%). Tectal plate lesions were noted in 4/12 individuals (33.3%), while central midbrain lesions were present in 3/12 (25%) cases. Supratentorial white matter volume loss and faint periventricular signal alterations were seen in 4/12 individuals (33.3%). Additional findings included multiple basal ganglia lesions (3/12, 25%), signal alterations in the inferior olivary nuclei and/or medulla (3/12, 25%), central tegmental tracts (2/12, 16.6%) and cervical spinal cord (1/12, 8.3%). Diffusion-weighted imaging was performed in 10 individuals and 12/19 MRI studies: focal areas of restricted diffusion were depicted in 5/12 studies at the level of the basal ganglia, left frontal white matter, substantia nigra and periaqueductal grey matter. All focal lesions were small (≤5 mm). MR spectroscopy was performed in three individuals and 6/19 MRI studies: high lactate peaks were noted at the level of the basal ganglia in 5/6 studies. Head CT was performed in two individuals and showed no intracranial calcifications. Longitudinal brain MRIs were available in six cases (two studies in five cases; three studies in one subject), at a median age of 1.8 years (IQR, 1–2.7 years) at first imaging and of 4.5 years (IQR, 2.7–8.2 years) at last follow-up. These studies revealed a reduction of the signal alterations in the brainstem (*n* = 5), progressive cerebellar atrophy (*n* = 4) and basal ganglia signal alterations with atrophy (*n* = 1). Subjects with cerebellar atrophy were all older than 5 years of age.

### Genetic findings

**Figure 3:**
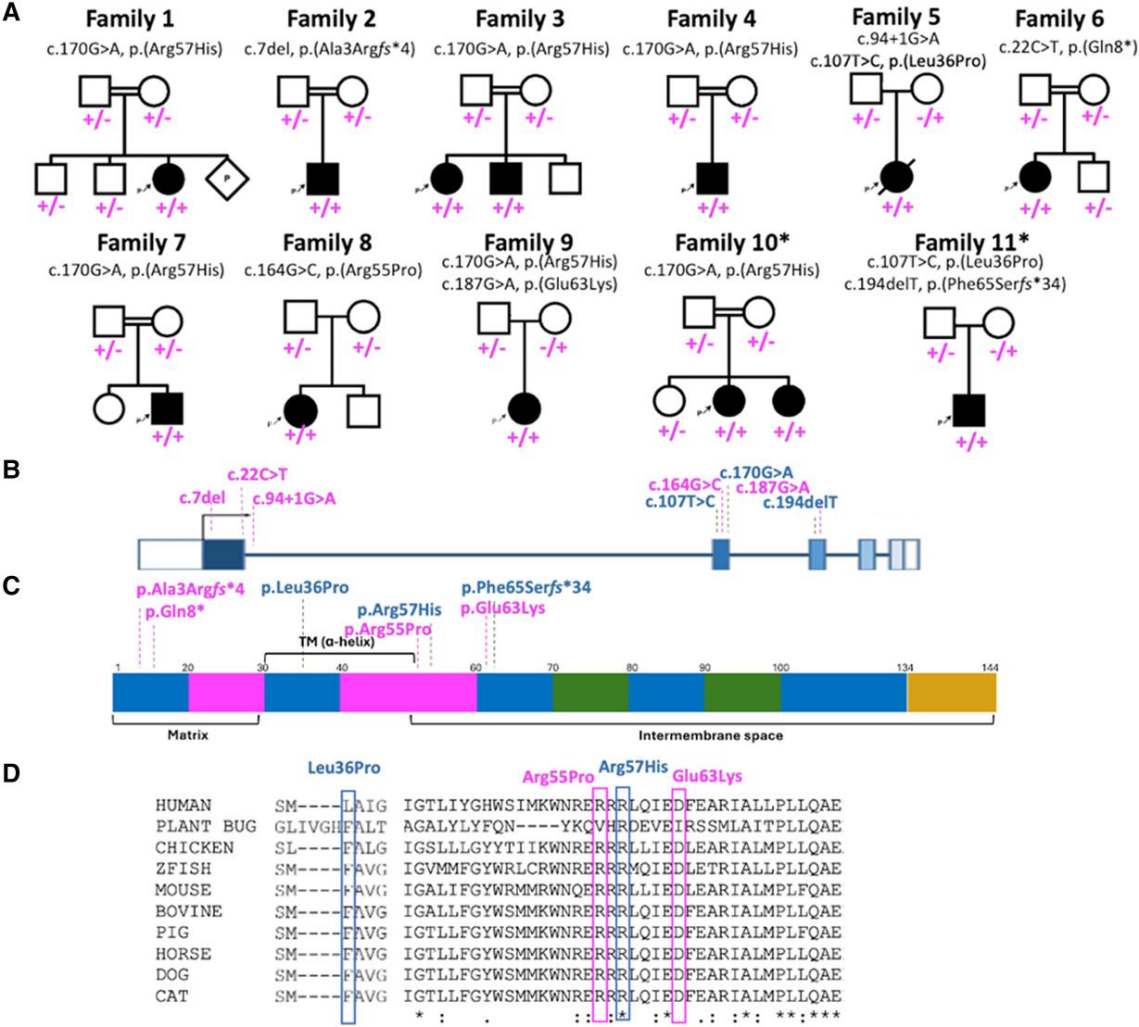
**Pedigrees with variant segregation, *NDUFA13* variant localizations on gene and protein schematics and variant conservation data**. (**A**) Family trees of the affected individuals with biallelic *NDUFA13* variants. Square = male; circle = female; black filled symbol = affected individual; white symbols = unaffected individuals; diagonal line = deceased individual. Double lines indicate consanguinity. The allele with the variant is indicated by the + sign. Wild-type allele is indicated by the −sign. * denotes already published families. (**B**) Schematic representation of the NDUFA13 gene showing the position of the variants (magenta denotes newly identified variants, blue denotes previously reported variants). (**C**) Diagram of the NDUFA13 protein with the position of the *NDUFA13* variants. Numbers indicate amino acids. (**D**) Interspecies alignment performed with Clustal Omega shows the level of conservation down to invertebrates on the missense *NDUFA13* variants. TM, transmembrane.

Molecular genetic findings included seven *NDUFA13* variants, including four missense and three predicted LOF variants. Families 1, 3, 4, 7 and 9 carried a previously reported (in Family 10) pathogenic homozygous variant in exon 2 of *NDUFA13* (NM_015965.7) c.170G > A, p.(Arg57His). We analysed the haplotypes of this variant in three unrelated families, and the results revealed no shared haplotype, indicating that this variant likely recurred independently. (Supplementary Fig. 1). Family 8 harboured a homozygous predicted-deleterious missense variant c.164G > C, p.(Arg55Pro). Family 9 harboured a heterozygous missense c.187G > A, p.(Glu63Lys) variant in *trans* with the recurrent *NDUFA13* c.170G > A variant. Interestingly, the *NDUFA13* c.187G > A, p.(Glu63Lys) variant identified in Family 9 in a heterozygous state was also found in a patient with limb malformation and skeletal dysplasia and congenital glaucoma, but in a homozygous state (Individual 14, Family 12). This patient underwent diagnostic exome sequencing, which revealed the *NDUFA13* c.187G > A, p.(Glu63Lys) as a rare homozygous variant. We considered this phenotype an outlier; therefore, we have not included this case in the description of the main cohort. However, the case report for this patient is available in the Supplementary material. Family 5 harboured an ultra-rare predicted deleterious missense *NDUFA13* c.107T > C, p.(Leu36Pro) variant, which was in *trans* with a splicing c.94 + 1G > A variant in *NDUFA13.* The missense c.107T > C, p.(Leu36Pro) variant was previously reported in the published Family 11. Family 2 harboured a novel homozygous frameshift variant c.7del, p.(Ala3ArgfsTer4) and Family 6 carried a homozygous protein-truncating variant that occurred at the beginning of the NDUFA13 amino acid sequence c.22C > T, p. (Gln8*). The variants segregated with the disease in all newly reported nine families (Fig. 3A) and are either absent or observed as heterozygous in extremely low frequencies in publicly available and private variant frequency databases (Supplementary Table 3). All three LOF *NDUFA13* variants reported in the present study are located within exon 1 or at the exon 1 -intron 1 boundary (Fig. 3B), and on the protein level, these LOF variants reside at the N-terminal matrix end or the C-terminal intermembrane space domain of the protein (Fig. 3C). The recurrent missense *NDUFA13* variant c.170G > A, p.(Arg57His) occurred at a highly conserved amino acid residue while the other three missense variants (Leu36, Arg55, Glu63) at moderately conserved residues, all with various *in silico* tools predicting a deleterious/damaging effect (Fig. 3D). All variants are within large regions of homozygosity. The homozygous splicing variant c.94 + 1G > A is predicted to affect splicing by alteration of the wild-type donor site between exons 1 and 2 (retrieved from SpliceAI server; Supplementary Fig. 2). The characteristics of the variants are summarized in Supplementary Table 3. No additional biallelic variants of likely pathogenic significance were identified in the cohort.

### Genotype–phenotype correlation

Although families carrying the recurring *NDUFA13* c.170G > A, p.(Arg57His) variant shared features such as infantile disease manifestation, slow disease progression rates, moderate-to-severe global global developmental delay and failure to acquire independent gait, there was a noticeable variation in the development of epileptic seizures, movement disorders and regression. Individuals with the LOF *NDUFA13* variants exhibited isolated motor delay with preserved cognitive function, whereas all homozygous carriers of the recurrent *NDUFA13* c.170G > A, p.(Arg57His) variant were cognitively impaired as part of their global developmental delay. Additionally, affected individuals with p.(Arg57His) tended to have slower disease progression rates when compared with the carriers of the LOF *NDUFA13* variants. Carriers of the other missense variant in *NDUFA13* presented with a milder phenotype. No clear correlation between genotype and neuroimaging features was observed. The homozygous carrier of the c.187G > A, p.(Glu63Lys) variant in *NDUFA13* presented with limb malformation and skeletal dysplasia and congenital glaucoma phenotype only, whereas the association of the same variant with the recurrent *NDUFA13* variant c.170G > A in a *trans*-state compound heterozygosity resulted in a neurological phenotype.

### The evaluation of the structural background of the variants

**Figure 4:**
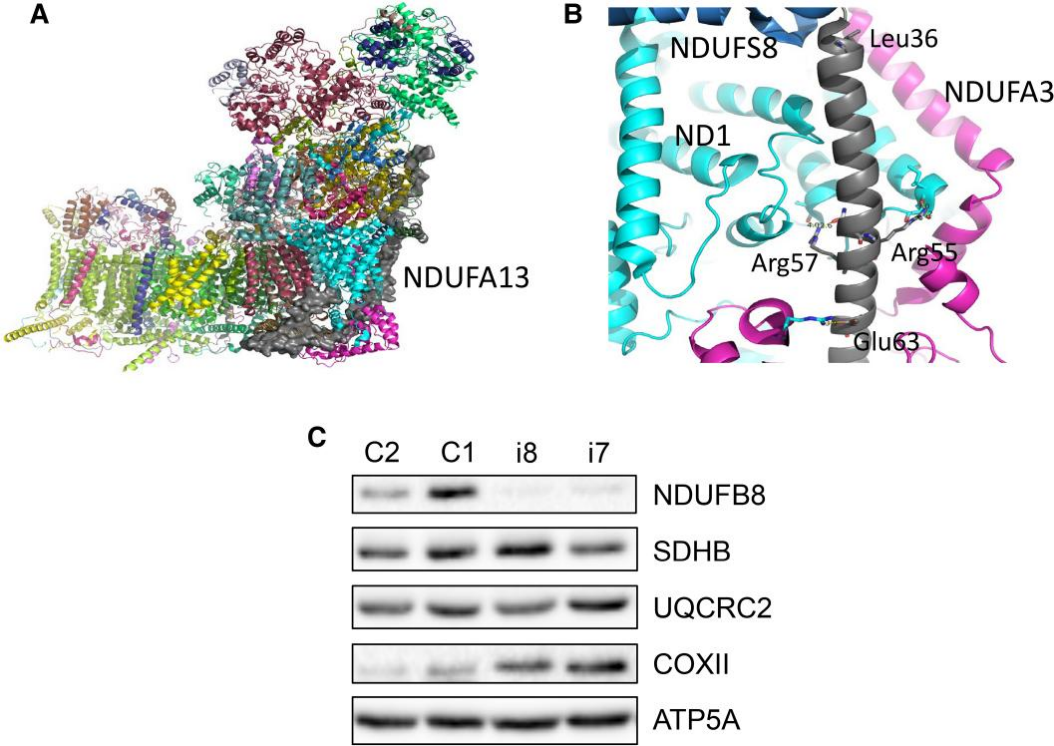
**Structural background of *NDUFA13* variants and biochemical assessment of fibroblasts from individuals with biallelic *NDUFA13* variants**. (**A**) Overview of human complex I (PDB 5XTD).^13^ View from the ‘back’, with membrane arm horizontal. Subunits are shown as cartoons and coloured differently. NDUFA13 subunit is highlighted as grey surface. (**B**) Interactions of NDUFA13 (grey) residues with neighbouring subunits ND1 (cyan), NDUFA3 (magenta) and NDUFS8 (blue). (**C**) Western blot analysis of OXPHOS components in individual (i7 and i8) and control (C1–2) fibroblasts (Supplementary Fig. 3 for source data). C, control; I, individual; NDUFS8, NADH-ubiquinone oxidoreductase iron-sulfur protein 8; NDUFA3, NADH-ubiquinone oxidoreductase subunit A3; NDUFB8, ND1, NADH dehydrogenase 1; NADH-ubiquinone oxidoreductase subunit B8; SDHB, succinate dehydrogenase complex, iron-sulfur subunit B; UQCRC2, ubiquinol-cytochrome c reductase core protein II; COXII, cytochrome c oxidase subunit 2; ATP5F1A, ATP synthase f1, subunit alpha.

We analysed the structural background of the altered residues and explored potential reasons for their harmful effects (Fig. 4A and B). Most of these variant residues are conserved across mammals, except for Leu36, which is replaced by Phe in most other species. The NDUFA13 subunit and its environment are also highly conserved (e.g. PDB 6ZKC^14^). Subunit NDUFA13 is tightly integrated within the complex at its ‘back’ at the junction of the peripheral and membrane arms (Fig. 4A). Here, it crosses the membrane from the matrix to IMS with one TM helix and interacts with many subunits, including core ND1 and NDUFS8, as well as accessory NDUFA3. All the altered residues interact with neighbouring subunits (Fig. 4B), likely contributing to the stability of this critical region, which connects the enzyme’s two arms. Leu36 forms hydrophobic contacts with NDUFS8 at the top of NDUFA13's TM helix. Its substitution with Pro weakens these contacts and is likely to disrupt the helical structure of NDUFA13 in this area. Arg55 and Arg57 are located at the C-terminal end of the NDUFA13 TM helix. Arg55 forms hydrogen bonds with the backbone oxygen of Ile311 and Ser312 from NDUFA3. Replacing Arg55 with Pro will disrupt both these bonds and the local helical structure. Arg57 forms hydrogen bonds with the backbone oxygen of Ser157 in the ND1 subunit and interacts with Asn54 in NDUFA13, locally stabilizing its TM helix. The Arg57 alteration to His is expected to destabilize this region. Subunit ND1 forms the main core of the junction between the two arms and also lines the quinone-binding cavity, which may explain the particular severity of the Arg57His variant. Lastly, Glu63 forms a hydrogen bond or weak salt bridge with Arg101 from the NDUFA3 subunit, likely stabilizing the exposed helices of NDUFA3 at the ‘heel’ of the complex. The substitution of Glu63 with Lys will disrupt this interaction, destabilizing the area.

### Biochemical and western blot analysis of fibroblasts

Cultured fibroblasts were obtained from the proband of Family 6 (Individual 7) and Family 7 (Individual 8). Both individual’s fibroblast samples demonstrated a specific decrease in the activity of mitochondrial CI activity: Individual 7: 0.120 nmols NADH oxidized.min^−1^.unit citrate synthase^−1^, controls 0.197 ± 0.034 (mean ± SD, *n* = 8); Individual 8: 0.072 nmols NADH oxidized.min^−1^.unit citrate synthase^−1^, controls 0.197 ± 0.034 (mean ± SD, *n* = 8) Furthermore, western blot analyses of key OXPHOS components showed a marked decrease in the steady-state levels of NDUFB8, another important CI subunit, whilst subunits of complex II (SDHA), complex III (UQCRC2) and complex V (ATP5A) were relatively unchanged. We did also note an apparent increase, possibly as a compensatory mechanism, in the steady-state level of the complex IV subunit COXII in both individuals (Fig. 4C).

### Literature review

We compiled the results of an extensive literature review, uniformly collecting the clinical details of all reported patients with isolated CI deficiency caused by 43 disease genes encoding CI subunits and assembly factors (Supplementary Table 4). Our analysis has shown that the clinical presentation of CI deficiency is incredibly heterogeneous, but the most common phenotype includes neurodevelopmental delay and hypotonia, frequently associated with lactic acidosis. Based on the frequency of reported symptoms and signs, we were able to identify a discernible core phenotype for genes with many reported families and thorough phenotyping. For example, our analysis revealed that, in addition to neurodevelopmental delay and hypotonia, frequent signs of *NDUFV1-*related disease (OMIM no. 618225) include spasticity, seizures, nystagmus and respiratory insufficiency. Dystonia is a frequent feature of *NDUFS7* (OMIM no. 618224), *NDUFA9* (OMIM no. 618247), *NDUFA10* (OMIM no. 618243) and *NDUFAF5-*related diseases (OMIM no. 618238). The combination of dystonia and cerebellar ataxia is seen in *NDUFAF6* (OMIM no. 618239) and *NDUFA12-*related diseases (OMIM no. 618244), while cerebellar ataxia is common in *NDUFA1* (OMIM no. 301020). Spastic ataxia is a common feature of *NDUFA13* (OMIM no. 618249) and *NUBPL* (OMIM no. 618242). Cardiomyopathy is frequently seen in *NDUFB11* (OMIM no. 301021), *NDUFS8* (OMIM no. 618222), *NDUFV2* (OMIM no. 618229) and *ACAD9* (OMIM no. 611126) defects. CI deficiency, associated with the following genes results in high mortality rates: *NDUFS1* (OMIM no. 618226), *NDUFS2* (OMIM no. 618228), *NDUFS4* (OMIM no. 252010), *NDUFAF1* (OMIM no. 618234) and *MT-ND3* (OMIM no. 516002).

## Discussion

Isolated deficiency of CI is the most commonly identified biochemical defect in childhood-onset mitochondrial disease, accounting for approximately a third of all cases of OXPHOS disorders.^7^ CI deficiency is clinically heterogeneous, but most affected individuals develop symptoms during the first year of life and have a rapidly progressive disease course, resulting in a fatal outcome in childhood.^7,15,16^ The most prevalent clinical presentations of CI deficiency include Leigh syndrome, leukoencephalopathy and various early-onset neurodegenerative disorders.^7^ There are >40 disease genes encoding structural subunits and assembly factors associated with mitochondrial CI deficiency.^17^ So far, no definitive correlation has been identified between the clinical phenotype and defects in CI subunits and assembly factors.^18^ However, it is important to consider that most genes leading to CI deficiency have been reported in only a small number of patients. This, combined with the variable degree of phenotyping among these patients, prevents us from seeing a full clinical spectrum for each gene associated with CI deficiency and from establishing a genotype–phenotype correlation. This was confirmed by our literature review, as we were able to suggest a core clinical phenotype specifically for those isolated CI deficiency–causing genes that had multiple reported families and detailed phenotyping. The purpose of our literature review was to identify a potential genotype–phenotype correlation and determine the core phenotype for each isolated CI deficiency–associated gene that might aid clinicians and medical geneticists in diagnosis.

Given the typically heterogeneous clinical presentation of CI deficiency, the phenotype of the previously reported individuals with biallelic variants in *NDUFA13* ranged from an infantile-onset slowly progressive encephalopathy with severe optic neuropathy, retinal dysfunction and prolonged survival without extra-neurological involvement in one family to Leigh syndrome in another. Isolated CI enzyme defect was found in skin fibroblast and muscle samples from three individuals.^8,9^ Our cumulative phenotypic analysis of nine new and two previously reported families suggested that the phenotype of defective *NDUFA13* encompasses mostly infantile-onset and non-rapidly progressing neurological impairment on the background of severe-to-profound and moderate global and isolated developmental delay. Spasticity, cerebellar ataxia, frequent movement disorders, optic nerve atrophy and subtle neuroimaging features related to Leigh syndrome with increased lactate were noted in most affected individuals. In particular, the most affected region was the substantia nigra as previously reported in other disorders of the oxidative phosphorylation system,^19^ followed by the periaqueductal grey matter, central midbrain, pons, medulla and basal ganglia with possible associated small areas of restricted diffusion. Notably, basal ganglia involvement was less frequent in subjects with *NDUFA13* variants compared to other CI deficiency disorders, i.e. 25% versus 90% of cases reported by Lebre *et al*.^20^ Moreover, while cerebellar cortical involvement is more common in Leigh syndrome due to variants in mtDNA genes,^19,20^ we found mild cerebellar atrophy in about 40% of *NDUFA13* cases after the age of 5 years. Finally, differently from other CI nuclear gene variants,^19-21^ cavitating leukoencephalopathy was not present in our series and white matter signal alterations were subtle and non-specific in all cases.

The sole limb malformation and skeletal dysplasia and congenital glaucoma phenotype in a patient with the homozygous *NDUFA13* c.187G > A, p.(Glu63Lys) variant could suggest that this variant is most probably hypomorphic. Although this missense variant has consistently been predicted to be deleterious and damaging in several *in silico* tools and has high GERP (4.5) and CADD (25.5) scores, in contrast to other *NDUFA13* variants reported in our study, its allele frequency in gnomAD Version 4 is 0.00003904, making an overall of 63 alleles out of 1 613 838. The variant is enriched within South Asian and European (non-Finish) populations. Hypomorphic variants are typically associated with milder clinical presentations due to only partial loss of normal gene function^22,23^ and require the presence of another pathogenic variant on the opposite allele to express a phenotype. Although some studies suggest that mitochondrial diseases could primarily manifest with skeletal phenotypes^24^ and congenital glaucoma,^25^ the role of the *NDUFA13* c.187G > A, p.(Glu63Lys) variant in the limb malformation and skeletal dysplasia and congenital glaucoma phenotype observed in Family 12 remains unclear.

Akin to most of the CI-deficient patients, the present cohort did not have dysmorphic features and were non-syndromic, with mostly normal pregnancy and birth parameters.^26^

In contrast to other CI deficiency syndromes, a slow disease progression with longer survival was suggested in previous *NDUFA13* reports suggesting a possible antiapoptotic effect of defective *NDUFA13*, the function mentioned previously.^8,9^ Interestingly, slow disease progression in our nine new families presenting with a neurological phenotype was in concordance with the previous reports highlighting the assumption that biallelic variants in *NDUFA13* might have less severe functional consequences than variants in other genes associated with CI deficiency.^8,9^ Alternatively, the broad range of *NDUFA13* functions might have influenced the rates of disease progression.^8,9^

Our protein modelling data suggest that the missense NDUFA13 variants reported in this study are likely to weaken the tight interaction network of several subunits at the critical junction between the two arms of the complex. This weakening could lead to defective assembly and a reduction in the amount of functional complex. The mechanism that couples redox reactions with proton pumping in CI relies on large, coordinated conformational changes around the quinone-binding site, located at the interface between the peripheral and membrane arms.^14,27^ Any destabilization of this interface due to alterations in NDUFA13 will result in reduced activity due to suboptimal coupling. The overall consequence of defective assembly and impaired coupling would be a diminished contribution of CI to mitochondrial energy production.

Similar to the previous reports of affected individuals with biallelic variants in *NDUFA13*, fibroblasts in the present study showed a marked decrease in NDUFA13 protein as well as other CI subunits and a decrease in the assembly of the CI holoenzyme.^8,9^ Individual 8, harbouring the same *NDUFA13* c.170G > A, p.(Arg57His) variant as previously reported, had some residual NDUFA13 protein levels, which is in accordance with the reported individuals.^8^ Interestingly, both affected individuals also displayed an increase in steady-state levels of complex IV protein subunits and an increase in complex IV activity. A previous study on Individual 11 also saw an increase of complex IV by BN-PAGE.^9^

Overall, our study expands the phenotypic and genetic spectrum of *NDUFA13*-related CI deficiency based on data from 13 affected individuals. The description of additional cases will further advance our understanding of *NDUFA13* defects in humans.

## Supplementary Material

fcae453_Supplementary_Data

## Data Availability

The authors have not generated any codes for this work. As such, there are no datasets or code repositories associated with this study.
