# Supplementary material for: Biallelic NDUFA13 variants lead to a neurodevelopmental phenotype with gradual neurological impairment
Source: Brain Commun. 2024 Dec 17;7(1):fcae453. doi: 10.1093/braincomms/fcae453 (PMC11832047; doi:10.1093/braincomms/fcae453)
Supplement: fcae453_Supplementary_Data [file fcae453_Supplementary_Data.zip › Supplementary Table 3.docx]

|  | **Subject ID** | **Probands F1-II:1, F3-II:3, II:4, F4-II:5, F7-II:8, F9-II:1, F10-II:12, II:13** | **Proband F2-II:2** | **Proband F5-II:6** | **Proband F6-II:7** | **Proband F8-II:9** | **Proband F9-II:10, Proband F- 12** | **Proband F5-II:6, Proband F11-II:14** | **Proband F11-II:14** |
| --- | --- | --- | --- | --- | --- | --- | --- | --- | --- |
| **Variant annotation** | GRCh38/hg38  Position, chr19.  (DNA change) | g.19526257G>T | g.19516245Gdel | g.19516333G>A | g.19516260C>T | g.19526251G>C | g.19527294G>A | g.19526194T>C | g.19527300Tdel |
|  | cDNA change (NM_015965.7) | c.170G>A | c.7del | c.94+1G>A | c.22C>T | c.164G>C | c.187G>A | c.107T>C | c.194delT |
|  | Protein change | p.(Arg57His) | p.(Ala3Arg*fs**4) | - | p.(Gln8*) | p.(Arg55Pro) | p.(Glu63Lys) | p.(Leu36Pro) | p.(Phe65Ser*fs**34) |
|  | Zygosity | Hom | Hom | Hom | Hom | Hom | Hom | Het | Het |
|  | Variant type | Missense | Frameshift | Splicing | Stopgain | Missense | Missense | Missense | Frameshift |
|  | dbSNP ID | rs752513525 | - | rs779602473 | rs777712324 | - | rs773496318 | rs2061098856 | rs2061105702 |
|  | Variant seen in family | F1, F3, F4, F7, F9, F10 | F2 | F5 | F6 | F8 | F9 | F11 | F11 |
| **Allele frequencies (PM2)** | gnomAD v4  (highest subpopulation) | 0.000009294 | - | 0.00004339 | 0.000006570 | - | 0.00003904 | 0.000001859 | 6.842e-7 |
|  | gnomAD v2.1.1  (highest subpopulation) | 0.00006436 | - | 0.00006436 | - | - | - | - | - |
|  | Frequency in ensembl browser | < 0.01 | NA | < 0.01 | < 0.01 | NA | < 0.01 | < 0.01 | NA |
|  | Iranome | - | - | - | - | - | - | - | - |
|  | GME Variome | - | - | - | - | - | - | - | - |
|  | GeneDx | <0.0001 | NA | <0.001 | <0.00001 |  | <0.0001 | NA | <0.001 |
|  | UKBB | 3/537492, 5.58148E-06 | - | - | - | - | 9/735926, 1.22295E-05 | - | - |
|  | Frequency in in-house database‡ | 1 het/~24000 | - | - | - | - | - | 1 het/~24000 | - |
| ***In silico* predictions (PP3)** | GERP | 4.58 | - | - | 5.3 | 4.9 | 4.55 | 5.08 | - |
|  | CADD | 28 | - | 34 | 40 | 26.3 | 25.5 | 25.6 | - |
|  | Polyphen-2 | 0.998 (PD) | - | - | - | 0.915 (PD) | 0.462 (PD) | 0.752 (PD) | - |
|  | SIFT | 0 (D) | - | - | - | 0.01 (D) | 0.03 (D) | 0 (D) | - |
|  | Provean | -4.067 (D) | -2.036 (N) | - | -8.028 (D) | -4.49 (D) | -3.97 (D) | -4.44 (D) | - |
|  | MutationTaster | 0.999 (D) | 1 (D) | D | 1 (D) | 0.9893 (B) | 0.999 (D) | 0.999 (D) | - |
|  | ACMG Classification | Pathogenic (PS1, PM2, PP1, PP2, PP3, PP4) | Likely pathogenic (PM2, PP3, PP4) | Likely pathogenic (PM2, PP3, PP4) | Pathogenic (PM2, PP3, PP4) | VUS (PP3, PM2) | VUS (PP3, PM2, BP3) | Likely pathogenic (PP3, PP5, PS3, PM2) | VUS (PP5, PS3, PM2) |

**Supplementary Table 3.** *NDUFA13* biallelic variants identified in the cohort.

F, family; Hom, homozygous; het, heterozygous; NA, not identified/absent in the database; D, deleterious, PD, probably deleterious; N, neutral.
