# Supplementary material for: Biallelic NDUFA13 variants lead to a neurodevelopmental phenotype with gradual neurological impairment
Source: Brain Commun. 2024 Dec 17;7(1):fcae453. doi: 10.1093/braincomms/fcae453 (PMC11832047; doi:10.1093/braincomms/fcae453)
Supplement: fcae453_Supplementary_Data [file fcae453_Supplementary_Data.zip › Supplementary_material.pdf]

SUPPLEMENTARY MATERIAL

SUPPLEMENTARY METHODS

WB supplementary methods

Immunoblotting was carried out using primary antibodies against various OXPHOS subunits all used at a dilution of 1 in 1000: NDUFB8 [Abcam ab110242], SDHB [abcam ab14714], UQCRC2 [Abcam ab14745], MT-CO1 [Abcam ab14705], MT-CO2 [abcam ab110258] and ATP5A [Abcam ab14748]) followed anti-mouse HRP-conjugated secondary antibodies (Dako: P0260 1 in 2000 dilution).

SUPPLEMENTARY CASE REPORTS

Family 1

The proband is a 2 years and 7 months old girl born full-term to a consanguineous Iranian family. Her prenatal history was unremarkable; however, later she presented failure to thrive and from the age of 8 months the signs of psychomotor retardation had become evident. She sat unsupported from the age of 2.5 years, started babbling from 1.5 years old, and has not acquired independent ambulation yet. Her body weight and height were at the 2nd percentile at the age of 2.5 years. From the age of 2 years, she developed abnormal eye movements. Her parents have not noticed any developmental regression. Upon examination, she was found to have an intellectual disability, limited ocular contact, and limited communication. There were slow saccadic eye movements and no signs of optic atrophy were present on fundoscopy. The hearing seemed to be preserved with normal brain stem auditory evoked response at the age of 1.5 years. She had poor head control accompanied by axial and peripheral hypotonia. Hyperreflexia, non-progressive spasticity, and truncal ataxia were present. She was mildly dyskinetic and choreoform movements were noticeable in the facial and limb muscles. Her hyperkinetic movement disorders have been gradually improving. She had little speech with the elements of dysarthria. No signs of diabetes and impaired renal and cardiac function were found. Her brain MRI at 18 months of age showed small symmetric substantia nigra lesions and periaqueductal gray matter signal alterations.

Family 2

The proband is a 3.3-year-old boy born full-term after uneventful pregnancy to consanguineous Iranian parents. His early neonatal measurements were within normal limits. Until age 1 years, he had gastroesophageal reflux. He sat unassisted at 1-year-old, walked at 2 years, and by age 1 was able to speak 10 words. He manifested with hypotonia and rapid developmental regression starting from the age of 2 years. This resulted in failure to thrive and loss of ambulation. From the age of 3 years, he 2 developed daily recurring myoclonic seizures responsive to clobazam. His EEG showed scattered sharp waves. At this last clinical follow-up at 3 years and 4 months of age, he was microcephalic with weight and height below 5th centile. He displayed limited communication with preserved ocular contact, left abducens nerve paresis, and slow saccades with normal eye fundi examination. His neuromuscular examination was remarkable with axial and peripheral hypotonia, and limb spasticity with hyperreflexia and extensor plantars. He is currently unable to walk and has dysarthric and poor speech. Movement disorders expressed included waxing and waning choreoathetoid movements of the limbs and face, dyskinesia, bradykinesia, and truncal ataxia. His serum lactate was elevated to 29 mg/dl with normal lactate levels in CSF and normal serum alanine. Brain MRI performed at 24 months of age showed bilateral small lesions in the basal ganglia (putamen, globi pallidi and caudate nuclei), substantia nigra and central midbrain with focal areas of restricted diffusion and cavitations. Follow-up brain MRI at 31 months showed increased confluent basal ganglia lesions with atrophy, reduction of the substantia nigra signal alterations, and mild reduction of periventricular white matter volume with enlargement of the cerebral subarachnoid spaces.

Family 3

The proband is an 11-year-old female born full-term to consanguineous parents of Pakistani origin. She has a 9-year-old affected brother. Her disease started at 3 months old with failure to thrive, diarrhea, and seizures. This had slowly progressed to severe psychomotor retardation. She has failed to acquire neck control, gaze fixation, and speech. She is not able to sit, stand and walk independently. Recurrent respiratory tract infections and respiratory distress have been her main medical issues. On examination,

she had infantile-onset abnormal eye movements, optic atrophy, and nystagmus. No sensorineural deafness was reported. Hyperreflexia, severe choreoathetoid movements of the limbs and face and extensor plantars were present. Although, no signs of appendicular hypotonia or spasticity were found on examination. She had tonic and/or clonic seizures with each episode lasting for 5-7 minutes. The maximum frequency of seizure attacks reached 1-2 per week. Her seizures are controlled by polytherapy including levetiracetam, valproic acid, and clonazepam. She is currently alive and does not have gastrointestinal issues, diabetes, renal problems, or cardiac pathology. Brain MRI performed at 3 years of age revealed optic nerve atrophy associated with bilateral basal ganglia lesions (globi pallidi) and signal alterations in the periaqueductal gray matter, tectal plate, and cerebellar dentate nuclei. At follow-up brain MRI at 9 years of age, there were new substantia nigra signal alterations, mild loss of WM volume, and cerebellar atrophy. There were no calcifications at brain CT.

Her affected brother presented with developmental delay and failure to thrive. He could sit unsupported by 8 months and stand with support by 9 months of age. At 9-month-old he manifested recurrent seizures with febrile illness and diarrhea followed by the regression of achieved milestones. He had rapidly lost achieved milestones within a period of one month. Since then recurrent tract infections and constipation have been the main medical problem. Currently, he is non-verbal, cannot understand any commands or gestures, and is not able to ambulate independently. Findings from his physical examination were similar to his sister's expect for additional signs of dyskinesia. In addition to tonic-clonic seizures, he had myoclonic seizures and his epilepsy is currently controlled by levetiracetam, valproic acid, and clonazepam. Seizure frequency and duration were similar to his sister's features. Brain MRI at age 7 years showed bilateral optic nerve atrophy associated with reduced cerebral white matter volume with faint periventricular signal alterations, enlarged CSF spaces, dentate and inferior olivary nuclei signal alterations and mild cerebellar atrophy. Brain CT revealed no calcifications.

Family 4

The proband is a 2 years and 8 months old male, a product of uneventful full-term pregnancy. His parents are first-cousins from Iraq. He presented with a motor delay, which came to the attention from

the age of 8 months when he failed to acquire unassisted sitting. Later this developed to the failed acquisition of speech, walking, and crawling skills. Currently, he ambulates by pushing with his legs off the floor when lying supine. He had a history of constipation, gastroesophageal reflux, and neuroirritability. He did not have epilepsy but a history of breath-holding spells was reported.

At his last examination performed at 2 years and 7 months old, his growth parameters were normal but he had severe intellectual disability, normal hearing, bilateral optic atrophy accompanied with nystagmus, and severe axial hypotonia. His nystagmus and reduced visual acuity were noted from the age of 19 months. He had limited ocular contact and was non-verbal. Laboratory tests revealed elevated serum lactate 4.1 mmol/L (16 months old), and 2.9 mmol/L (2 years 5 months old). Brain MRI at 23 months of age showed bilateral optic nerve atrophy associated with reduced cerebral white matter volume and signal alterations, prominent cerebral CSF spaces, dentate nuclei and inferior olivary nuclei signal alterations. No lactate peaks were found at MR spectroscopy performed at the level of the basal ganglia.

Family 5

The affected individual is a girl born to unrelated Caucasian parents. She was born full-term after an uneventful pregnancy and delivery with borderline newborn measurements (weight – below 5th percentile, height- 25th percentile, and head circumference - 4th percentile). At 12 months of age, she presented with failure to thrive and developmental delay followed by developmental regression. She sat independently by 12 months of age, started walking from 2 years old, and started using first words from 15 months old. Since the disease onset, her main medical problem was hypotonia. She reported having respiratory distress, myopia, and constipation. She was found to have signs compatible with Leigh's disease and mitochondrial complex I deficiency. By the age of 5 years, she lost independent ambulation as a result of motor regression and died at age 6 years. Upon her last examination at 6 years old, she had borderline microcephaly, with no signs of intellectual disability but limited communication. She could maintain ocular contact and had a good interaction with family members. There were ptosis, dysarthria, axial hypotonia, and progressive spasticity coupled with truncal ataxia, global hypokinesia,

hyperreflexia, and peripheral neuropathy among the neurological findings. She had neither epilepsy nor sensory deficit. Laboratory tests revealed lactic acidosis (4.9mmol/L) and elevated serum alanine (734 mmol/L). Muscle histochemistry showed increased oxidative reactivity of cytochrome C oxidase and the analysis of respiratory chain enzymes revealed mitochondrial complex I deficiency. Brain MRI performed at the age of 30 months showed bilateral optic nerve atrophy associated with small symmetric lesions in the substantia nigra, tectal plate, central midbrain, central tegmental tracts, and cervical spinal cord (at the level of the bulbo-medullary junction). A small area of restricted diffusion was noted at the level of the left periventricular frontal white matter.

Family 6

The proband is a 4-year-old girl born full term following unremarkable pregnancy and delivery to consanguineous Arab parents. Her neonatal period was unremarkable but later some developmental delay was noticed. She sat unsupported at 8 months, walked independently from 23 months, and started using first words from 18 months of age. At 4 months she manifested nystagmus. From 6 months reduced vision-related to optic neuropathy was revealed. No history of epileptic seizures or hearing impairment was reported. At the last follow-up at the age of 4 years and 9 months, her growth parameters were normal. She had limited eye contact but did not have an intellectual disability and was able to actively interact with her family members. Convergent strabismus, peripheral hypotonia with truncal ataxia, choreoathetoid, and dyskinetic movements of the limbs and face were the main neurological findings. Her movement disorders have remarkably improved over the years. The rest of the physical examination was normal. Her serum lactate levels were normal but CSF lactate was mildly elevated to 2.8 mmol/L. Fibroblast studies revealed reduced levels of NDUFA13 along with other Complex I subunit NDUFB8 compared to controls. BN-PAGE on patient's fibroblasts revealed severely reduced levels of Complex I assembly compared to controls. Brain MRI performed at 9 months showed bilateral optic nerve atrophy associated with bilateral substantia nigra, periaqueductal gray matter and central tegmental tracts signal alterations. Bilateral foci of restricted diffusion were noted at the level of the substantia nigra. Follow-up brain MRI at 3 years of age revealed new basal ganglia (putamen and

caudate) lesions with high lactate peaks at MR spectroscopy. The midbrain lesions were reduced and mild cerebellar atrophy involving the lateral cerebellar hemispheres was evident.

Family 7

The proband 8.5-year-old boy born full-term to consanguineous parents of Iranian decent. His prenatal and early neonatal features were unremarkable. His dissease manifested with central hypotonia, global developmental delay, and seizures followed by speech regression starting from the age of 9 months. The disease progressed slowly with failure to thrive, unachieved motor milestones and speech.

At the most recent follow-up he was age 8 years old and presented with microcephaly, intellectual disability, and weight and height below the 5th percentile. He had nystagmus, slow saccades, axial hypotonia, poor head control, and spasticity. He was non-ambulatory and non-verbal. His epileptic seziures were mostly myoclonic and recurred every week with valproic acid and nitrazepam. EEG showed diffuse paroxysmal epileptic form discharge. Among non-neurological signs, gastroosophageal reflux disease was reported. His renal, liver, and cardiac functions as well as CSF lactate, plasma and urine amino acids and organic acids leves were normal. Brain MRI at 9 months of age was normal, while follow-up MRI at 18 months revealed bilateral substantia nigra, periaqueductal gray matter and dentate nuclei signal alterations. Additional faint periventricular white matter volume signal alterations were seen.

Family 8

The patient, a 3-year-old Indian male, presented with symptoms of ataxia at 18 months of age, followed by developmental delay and failure to thrive. Physical examination revealed axial and peripheral hypotonia, hyperreflexia, dysarthria, and truncal ataxia. Ocular assessment demonstrated nystagmus, reduced visual acuity, and bilateral optic disc pallor. Laboratory investigations revealed elevated serum lactate levels indicative of lactic acidosis. Brain MRI performed at 3 years of age revealed bilateral

optic nerve atrophy associated with symmetric lesions in the substantia nigra, periaqueductal gray matter, and central midbrain. Faint signal alterations were noted at the level of the supratentorial white matter and cerebellar dentate nuclei. Small foci of restricted diffusion were detected in the periaqueductal gray matter and central midbrain.

Family 9

The patient, a Middle Eastern female, was born at term after an uneventful gestation period, with a birth weight of 3 kg and a birth length of 49 cm. She achieved sitting at 7 months, walking at 14 months, and her first words/language abilities at 9 months of age. Upon initiating walking, she exhibited poor balance. Her developmental milestones were within normal limits, and no regression was noted. Neurological examination revealed intermittent truncal ataxia and ataxic gait. Dysarthria was absent, and sensory examination was normal.

Laboratory investigations showed slightly elevated serum lactate levels, while genetic testing uncovered two trans-acting rare predicted-deleterious heterozygous variants in *NDUFA13* (NM_015965.7) c.170G>A, p.(Arg57His) and c.187G>A, p.(Glu63Lys).

Brain MRI performed at 6 months of age was reported as normal. Brain MRI at 24 months of age revealed bilateral signal changes with restricted diffusion in white matter tracts in the posterior medulla associated with bilateral lesions in the substantia nigra.

Family 12

A female patient, 14 years old presented to the Limb Malformation and Skeletal Dysplasia (LMSD) clinic due to the associated skeletal deformities and congenital Glaucoma. She was born to consanguineous parents who have a history of three abortions and two older healthy children. At birth she had congenital glaucoma, bilateral talipes, right knee dislocation, and right patellar sublaxation and required many operations. Walking was achieved at the age of 2 years while her mentality was within

the average. On examination, she had upward slanting of palpebral fissure, scoliosis, tilted pelvis, and right knee deformity. Skeletal survey revealed shallow acetabulum, small head of femurs, bilateral small flat irregularly ossified around knee epiphysis, bilateral bifid knee epiphysis, lateral displacement of right patella, and mild metaphyseal sclerosis. IQ test was equal to 102. Hearing test and echo heart were normal. Since the patient had an average cognitive function brain MRI has not been pursued. The delay in walking is attributable to the knee joint dislocation. The patients did not have any typical features of CI deficiency; hence respiratory chain enzyme testing has not been done.

Supplementary Figures

Supplementary Figure 1. Haplotype analysis.

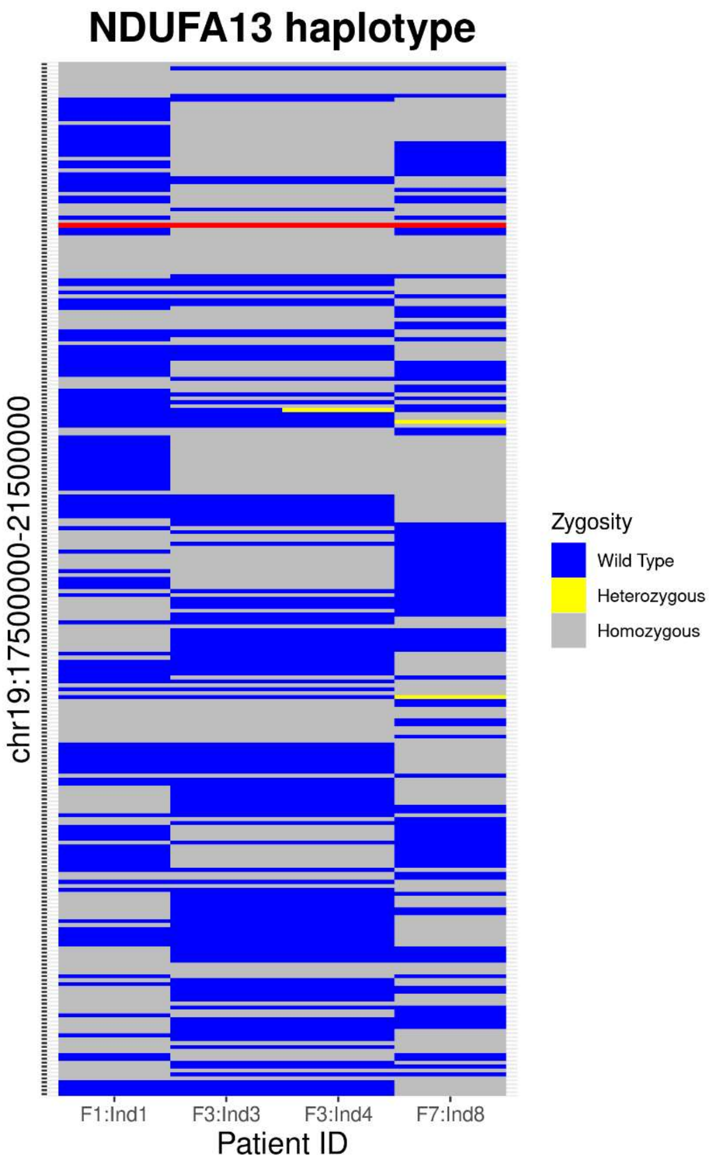

Haplotype plot of the shared *NDUFA13* (NM_015965.7) c.170G>A variant in 3 families. All 4 patients are homozygote in the locus, however, the pattern of variants flanking the pathogenic *NDUFA13* variant differs among these 3 families.

Supplementary Figure 2. Splice AI output for the splicing *NDUFA13* c.94+1G>A variant.

19-19516333-G-A
UCSC, gnomAD

NDUFA13
OMIM, GTEx, gnomAD, ClinGen, Ensembl, Decipher, GeneCards

Acceptor Loss	0.00	
Donor Loss	0.99	-1 bp
Acceptor Gain	0.00	
Donor Gain	0.09	3 bp

UCSC, the University of California, Santa Cruz genome browser tool; OMIM, Online Mendelian Inheritance in Man; GTEx, Genotype-Tissue Expression project; bp, base pairs.

Supplementary Figure 3. Full-size and uncropped blots/gels.

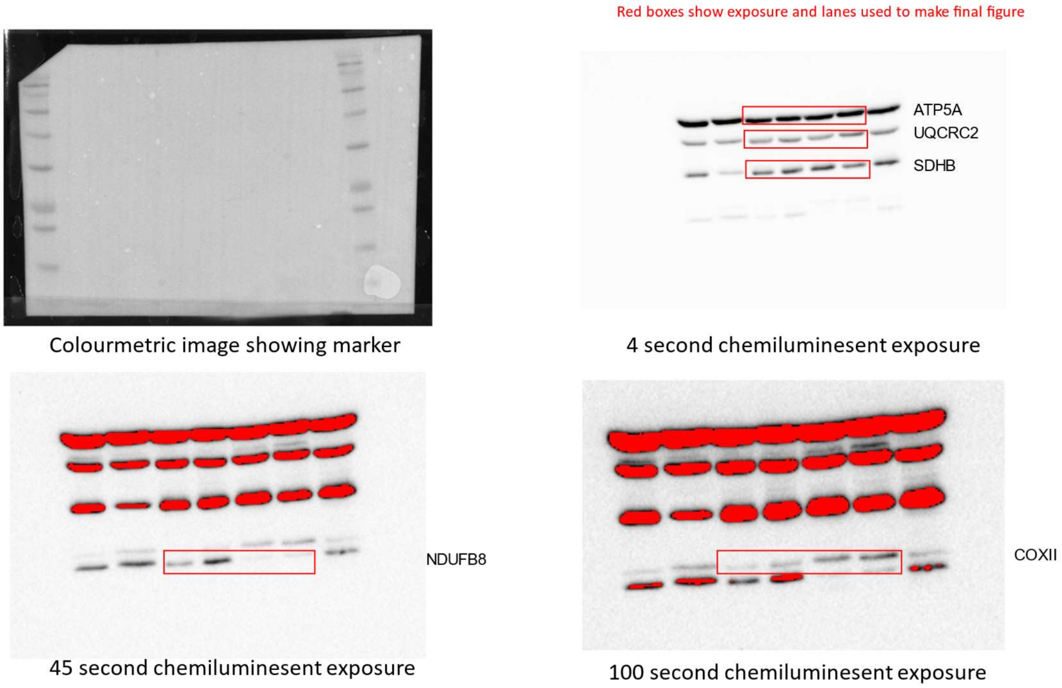

Colourmetric image showing marker

4 second chemiluminesent exposure

45 second chemiluminesent exposure

100 second chemiluminesent exposure

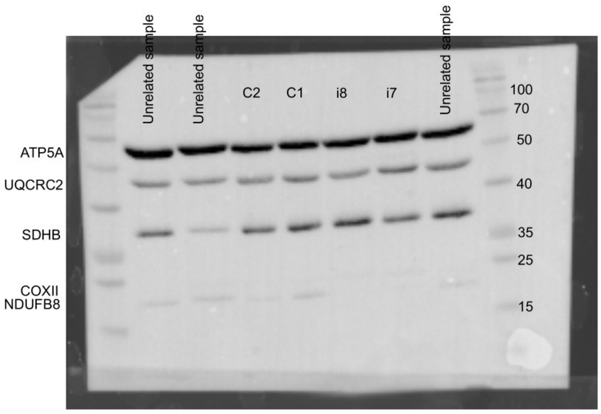

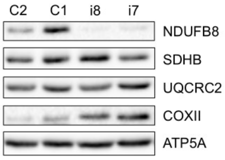

Merged image of colourimetric image and
4 second chemiluminesent exposure

Supplementary Table 1. Clinical features of the individuals with biallelic variants in *NDUFA13*. *Available as a separate Excel file.*

Supplementary Table 2. Extended clinical table**.** *Available as a separate Excel file.*

Supplementary Table 3. Characteristics of the *NDUFA13* variants identified in the cohort. *Available as a separate Word file.*

Supplementary Table 4. Clinical features of complex I deficiency associated disease genes. *Available as a separate Excel file.*

Supplementary literature

Literature from Supplementary Table 1

Poole OV, Pizzamiglio C, Murphy D, et al. Mitochondrial DNA Analysis from Exome Sequencing Data Improves Diagnostic Yield in Neurological Diseases. Ann Neurol. 2021 Jun;89(6):1240-1247.

Kaiyrzhanov R, Mohammed SEM, Maroofian R, et al. Bi-allelic LETM1 variants perturb mitochondrial ion homeostasis leading to a clinical spectrum with predominant nervous system involvement. Am J Hum Genet. 2022 Sep 1;109(9):1692-1712.

Guillen Sacoto MJ, Tchasovnikarova IA, Torti E, et al. De Novo Variants in the ATPase Module of MORC2 Cause a Neurodevelopmental Disorder with Growth Retardation and Variable Craniofacial Dysmorphism. Am J Hum Genet. 2020 Aug 6;107(2):352-363. d

Angebault C, Charif M, Guegen N, et al. Mutation in NDUFA13/GRIM19 leads to early onset hypotonia, dyskinesia and sensorial deficiencies, and mitochondrial complex I instability. Hum Mol Genet. 2015 Jul 15;24(14):3948-55.

Gonzalez-Quintana A, Garcia-Consuegra I, Belanger-Quintana A, et al. Novel NDUFA13 Mutations Associated with OXPHOS Deficiency and Leigh Syndrome: A Second Family Report. Genes (Basel). 2020 Jul 26;11(8).

Literature from Supplementary Table 4

NDUF-related genes:

1. Bénit, P., Chretien, D., Kadhom, N., de Lonlay-Debeney, P., Cormier-Daire, V., Cabral, A., Peudenier, S., Rustin, P., Munnich, A., & Rötig, A. (2001). Large-scale deletion and point mutations of the nuclear NDUFV1 and NDUFS1 genes in mitochondrial complex I deficiency. American journal of human genetics, 68(6), 1344–1352. https://doi.org/10.1086/320603

2. Martín, M. A., Blázquez, A., Gutierrez-Solana, L. G., Fernández-Moreira, D., Briones, P., Andreu, A. L., Garesse, R., Campos, Y., & Arenas, J. (2005). Leigh syndrome associated with mitochondrial complex I deficiency due to a novel mutation in the NDUFS1 gene. Archives of neurology, 62(4), 659–661. https://doi.org/10.1001/archneur.62.4.659

3. Distelmaier, F., Koopman, W. J., van den Heuvel, L. P., Rodenburg, R. J., Mayatepek, E., Willems, P. H., & Smeitink, J. A. (2009). Mitochondrial complex I deficiency: from organelle dysfunction to clinical disease. Brain : a journal of neurology, 132(Pt 4), 833–842. https://doi.org/10.1093/brain/awp058

4. Hoefs, S. J., Skjeldal, O. H., Rodenburg, R. J., Nedregaard, B., van Kaauwen, E. P., Spiekerkötter, U., von Kleist-Retzow, J. C., Smeitink, J. A., Nijtmans, L. G., & van den Heuvel, L. P. (2010). Novel mutations in the NDUFS1 gene cause low residual activities in human complex I deficiencies. Molecular genetics and metabolism, 100(3), 251–256. https://doi.org/10.1016/j.ymgme.2010.03.015

5. Ferreira, M., Torraco, A., Rizza, T., Fattori, F., Meschini, M. C., Castana, C., Go, N. E., Nargang, F. E., Duarte, M., Piemonte, F., Dionisi-Vici, C., Videira, A., Vilarinho, L., Santorelli, F. M., Carrozzo, R., & Bertini, E. (2011). Progressive cavitating leukoencephalopathy associated with respiratory chain complex I deficiency and a novel mutation in NDUFS1. Neurogenetics, 12(1), 9–17. https://doi.org/10.1007/s10048-010-0265-2

6. Kashani, A., Thiffault, I., Dilenge, M. E., Saint-Martin, C., Guerrero, K., Tran, L. T., Shoubridge, E., van der Knaap, M. S., Braverman, N., & Bernard, G. (2014). A homozygous mutation in the NDUFS1 gene presents with a mild cavitating leukoencephalopathy. Neurogenetics, 15(3), 161–164. https://doi.org/10.1007/s10048-014-0412-2

7. Björkman, K., Sofou, K., Darin, N., Holme, E., Kollberg, G., Asin-Cayuela, J., Holmberg Dahle, K. M., Oldfors, A., Moslemi, A. R., & Tulinius, M. (2015). Broad phenotypic variability in

patients with complex I deficiency due to mutations in NDUFS1 and NDUFV1. Mitochondrion, 21, 33–40. https://doi.org/10.1016/j.mito.2015.01.003

8. Men, L., Feng, J., Huang, W., Xu, M., Zhao, X., Sun, R., Xu, J., & Cao, L. (2022). Lip cyanosis as the first symptom of Leigh syndrome associated with mitochondrial complex I deficiency due to a compound heterozygous NDUFS1 mutation: A case report. Medicine, 101(34), e30303. https://doi.org/10.1097/MD.0000000000030303

9. Loeffen, J., Elpeleg, O., Smeitink, J., Smeets, R., Stöckler-Ipsiroglu, S., Mandel, H., Sengers, R., Trijbels, F., & van den Heuvel, L. (2001). Mutations in the complex I NDUFS2 gene of patients with cardiomyopathy and encephalomyopathy. Annals of neurology, 49(2), 195–201. https://doi.org/10.1002/1531-8249(20010201)49:2<195::aid-ana39>3.0.co;2-m

10. Tuppen, H. A., Hogan, V. E., He, L., Blakely, E. L., Worgan, L., Al-Dosary, M., Saretzki, G., Alston, C. L., Morris, A. A., Clarke, M., Jones, S., Devlin, A. M., Mansour, S., Chrzanowska-Lightowlers, Z. M., Thorburn, D. R., McFarland, R., & Taylor, R. W. (2010). The p.M292T NDUFS2 mutation causes complex I-deficient Leigh syndrome in multiple families. Brain : a journal of neurology, 133(10), 2952–2963. https://doi.org/10.1093/brain/awq232

11. Marin, S. E., Mesterman, R., Robinson, B., Rodenburg, R. J., Smeitink, J., & Tarnopolsky, M. A. (2013). Leigh syndrome associated with mitochondrial complex I deficiency due to novel mutations In NDUFV1 and NDUFS2. Gene, 516(1), 162–167. https://doi.org/10.1016/j.gene.2012.12.024

12. Ngu, L. H., Nijtmans, L. G., Distelmaier, F., Venselaar, H., van Emst-de Vries, S. E., van den Brand, M. A., Stoltenborg, B. J., Wintjes, L. T., Willems, P. H., van den Heuvel, L. P., Smeitink, J. A., & Rodenburg, R. J. (2012). A catalytic defect in mitochondrial respiratory chain complex I due to a mutation in NDUFS2 in a patient with Leigh syndrome. Biochimica et biophysica acta, 1822(2), 168–175. https://doi.org/10.1016/j.bbadis.2011.10.012

13. Rubrecht, A., Clapp, W., & Shenoy, A. (2020). Liver Pathology in Mitochondrial Complex I Deficiency from Bi-Allelic Mutations in NDUFS2: A Report of Findings at Autopsy. Fetal and pediatric pathology, 39(3), 259–262. https://doi.org/10.1080/15513815.2019.1651800

14. Bénit, P., Slama, A., Cartault, F., Giurgea, I., Chretien, D., Lebon, S., Marsac, C., Munnich, A., Rötig, A., & Rustin, P. (2004). Mutant NDUFS3 subunit of mitochondrial complex I causes Leigh syndrome. Journal of medical genetics, 41(1), 14–17. https://doi.org/10.1136/jmg.2003.014316

15. Pagniez-Mammeri, H., Lombes, A., Brivet, M., Ogier-de Baulny, H., Landrieu, P., Legrand, A., & Slama, A. (2009). Rapid screening for nuclear genes mutations in isolated respiratory chain complex I defects. Molecular genetics and metabolism, 96(4), 196–200. https://doi.org/10.1016/j.ymgme.2008.12.003

16. Haack, T. B., Haberberger, B., Frisch, E. M., Wieland, T., Iuso, A., Gorza, M., Strecker, V., Graf, E., Mayr, J. A., Herberg, U., Hennermann, J. B., Klopstock, T., Kuhn, K. A., Ahting, U., Sperl, W., Wilichowski, E., Hoffmann, G. F., Tesarova, M., Hansikova, H., Zeman, J., … Prokisch, H. (2012). Molecular diagnosis in mitochondrial complex I deficiency using exome sequencing. Journal of medical genetics, 49(4), 277–283. https://doi.org/10.1136/jmedgenet-2012-100846

17. Johnstone, T., Wang, J., Ross, D., Balanda, N., Huang, Y., Godfrey, R., Groden, C., Barton, B. R., Gahl, W., Toro, C., & Malicdan, M. C. V. (2020). Biallelic variants in two complex I genes cause abnormal splicing defects in probands with mild Leigh syndrome. Molecular genetics and metabolism, 131(1-2), 98–106. https://doi.org/10.1016/j.ymgme.2020.09.008

18. Samanta, D., Veerapandiyan, A., Burrow, T. A., & Gokden, M. (2021). Mitochondrial Ultrastructural Defects in NDUFS3-Related Disorder. Journal of pediatric neurosciences, 16(4), 299–302. https://doi.org/10.4103/jpn.JPN_182_20

19. Loeffen, J. L., Smeitink, J. A., Trijbels, J. M., Janssen, A. J., Triepels, R. H., Sengers, R. C., & van den Heuvel, L. P. (2000). Isolated complex I deficiency in children: clinical, biochemical and genetic aspects. Human mutation, 15(2), 123–134. https://doi.org/10.1002/(SICI)1098-1004(200002)15:2<123::AID-HUMU1>3.0.CO;2-P

20. Budde, S. M., van den Heuvel, L. P., Janssen, A. J., Smeets, R. J., Buskens, C. A., DeMeirleir, L., Van Coster, R., Baethmann, M., Voit, T., Trijbels, J. M., & Smeitink, J. A. (2000). Combined enzymatic complex I and III deficiency associated with mutations in the nuclear encoded NDUFS4 gene. Biochemical and biophysical research communications, 275(1), 63–68. https://doi.org/10.1006/bbrc.2000.3257

21. Petruzzella, V., Vergari, R., Puzziferri, I., Boffoli, D., Lamantea, E., Zeviani, M., & Papa, S. (2001). A nonsense mutation in the NDUFS4 gene encoding the 18 kDa (AQDQ) subunit of complex I abolishes assembly and activity of the complex in a patient with Leigh-like syndrome. Human molecular genetics, 10(5), 529–535. https://doi.org/10.1093/hmg/10.5.529

22. Bénit, P., Steffann, J., Lebon, S., Chretien, D., Kadhom, N., de Lonlay, P., Goldenberg, A., Dumez, Y., Dommergues, M., Rustin, P., Munnich, A., & Rötig, A. (2003). Genotyping microsatellite DNA markers at putative disease loci in inbred/multiplex families with respiratory chain complex I deficiency allows rapid identification of a novel nonsense mutation (IVS1nt -1) in the NDUFS4 gene in Leigh syndrome. Human genetics, 112(5-6), 563–566. https://doi.org/10.1007/s00439-002-0884-2

23. Iuso, A., Scacco, S., Piccoli, C., Bellomo, F., Petruzzella, V., Trentadue, R., Minuto, M., Ripoli, M., Capitanio, N., Zeviani, M., & Papa, S. (2006). Dysfunctions of cellular oxidative metabolism in patients with mutations in the NDUFS1 and NDUFS4 genes of complex I. The Journal of biological chemistry, 281(15), 10374–10380. https://doi.org/10.1074/jbc.M513387200

24. Anderson, S. L., Chung, W. K., Frezzo, J., Papp, J. C., Ekstein, J., DiMauro, S., & Rubin, B. Y. (2008). A novel mutation in NDUFS4 causes Leigh syndrome in an Ashkenazi Jewish family. Journal of inherited metabolic disease, 31 Suppl 2, S461–S467. https://doi.org/10.1007/s10545-008-1049-9

25. Leshinsky-Silver, E., Lebre, A. S., Minai, L., Saada, A., Steffann, J., Cohen, S., Rötig, A., Munnich, A., Lev, D., & Lerman-Sagie, T. (2009). NDUFS4 mutations cause Leigh syndrome with predominant brainstem involvement. Molecular genetics and metabolism, 97(3), 185–189. https://doi.org/10.1016/j.ymgme.2009.03.002

26. Assereto, S., Robbiano, A., Di Rocco, M., Rossi, A., Cassandrini, D., Panicucci, C., Brigati, G., Biancheri, R., Bruno, C., Minetti, C., Trucks, H., Sander, T., Zara, F., & Gazzerro, E. (2014). Functional characterization of the c.462delA mutation in the NDUFS4 subunit gene of mitochondrial complex I. Clinical genetics, 86(1), 99–101. https://doi.org/10.1111/cge.12248

27. Ortigoza-Escobar, J. D., Oyarzabal, A., Montero, R., Artuch, R., Jou, C., Jiménez, C., Gort, L., Briones, P., Muchart, J., López-Gallardo, E., Emperador, S., Pesini, E. R., Montoya, J., Pérez, B., Rodríguez-Pombo, P., & Pérez-Dueñas, B. (2016). Ndufs4 related Leigh syndrome: A case report and review of the literature. Mitochondrion, 28, 73–78. https://doi.org/10.1016/j.mito.2016.04.001

28. Lamont, R. E., Beaulieu, C. L., Bernier, F. P., Sparkes, R., Innes, A. M., Jackel-Cram, C., Ober, C., Parboosingh, J. S., & Lemire, E. G. (2017). A novel NDUFS4 frameshift mutation causes Leigh disease in the Hutterite population. American journal of medical genetics. Part A, 173(3), 596–600. https://doi.org/10.1002/ajmg.a.37983

29. Bris, C., Rouaud, T., Desquiret-Dumas, V., Gueguen, N., Goudenege, D., Barth, M., Bonneau, D., Amati-Bonneau, P., Lenaers, G., Reynier, P., Lebre, A. S., & Procaccio, V. (2017). Novel NDUFS4 gene mutation in an atypical late-onset mitochondrial form of multifocal dystonia. Neurology. Genetics, 3(6), e205. https://doi.org/10.1212/NXG.0000000000000205

30. González-Quintana, A., Trujillo-Tiebas, M. J., Fernández-Perrone, A. L., Blázquez, A., Lucia, A., Morán, M., Ugalde, C., Arenas, J., Ayuso, C., & Martín, M. A. (2020). Uniparental isodisomy as a cause of mitochondrial complex I respiratory chain disorder due to a novel splicing NDUFS4 mutation. Molecular genetics and metabolism, 131(3), 341–348. https://doi.org/10.1016/j.ymgme.2020.10.008

31. Vafaee-Shahi, M., Ghasemi, S., Beiraghi Toosi, M., Ashrafi, M. R., Badv, R. S., Tavasoli, A. R., & Tahernia, L. (2021). Bilateral horizontal gaze palsy in an 8-year-old girl: A rare case with NDUFS4 gene mutation. Clinical case reports, 9(9), e04748. https://doi.org/10.1002/ccr3.4748

32. Rouzier, C., Chaussenot, A., Fragaki, K., Serre, V., Ait-El-Mkadem, S., Richelme, C., Paquis-Flucklinger, V., & Bannwarth, S. (2019). NDUFS6 related Leigh syndrome: a case report and review of the literature. Journal of human genetics, 64(7), 637–645. https://doi.org/10.1038/s10038-019-0594-4

33. Spiegel, R., Shaag, A., Mandel, H., Reich, D., Penyakov, M., Hujeirat, Y., Saada, A., Elpeleg, O., & Shalev, S. A. (2009). Mutated NDUFS6 is the cause of fatal neonatal lactic acidemia in Caucasus Jews. European journal of human genetics : EJHG, 17(9), 1200–1203. https://doi.org/10.1038/ejhg.2009.24

34. Kirby, D. M., Salemi, R., Sugiana, C., Ohtake, A., Parry, L., Bell, K. M., Kirk, E. P., Boneh, A., Taylor, R. W., Dahl, H. H., Ryan, M. T., & Thorburn, D. R. (2004). NDUFS6 mutations are a novel cause of lethal neonatal mitochondrial complex I deficiency. The Journal of clinical investigation, 114(6), 837–845. https://doi.org/10.1172/JCI20683

35. Li, Y., Zhang, Y., Jiang, G., Wang, Y., He, C., Zhao, X., Liu, L., & Li, L. (2022). Case report: novel mutations of NDUFS6 and NHLRC2 genes potentially cause the quick postnatal death of a Chinese Hani minority neonate with mitochondrial complex I deficiency and FINCA syndrome. Medicine, 101(27), e29239. https://doi.org/10.1097/MD.0000000000029239

36. Gangfuß, A., Rating, P., Ferreira, T., Hentschel, A., Marina, A. D., Kölbel, H., Sickmann, A., Abicht, A., Kraft, F., Ruck, T., Böhm, J., Schänzer, A., Schara-Schmidt, U., Neuhann, T. M., Horvath, R., & Roos, A. (2024). A Homozygous NDUFS6 Variant Associated with Neuropathy and Optic Atrophy. Journal of neuromuscular diseases, 11(2), 485–491. https://doi.org/10.3233/JND-230181

37. Pronicka, E., Piekutowska-Abramczuk, D., Ciara, E., Trubicka, J., Rokicki, D., Karkucińska-Więckowska, A., Pajdowska, M., Jurkiewicz, E., Halat, P., Kosińska, J., Pollak, A., Rydzanicz, M., Stawinski, P., Pronicki, M., Krajewska-Walasek, M., & Płoski, R. (2016). New perspective in diagnostics of mitochondrial disorders: two years' experience with whole-exome sequencing at a national paediatric centre. Journal of translational medicine, 14(1), 174. https://doi.org/10.1186/s12967-016-0930-9

38. Koene, S., Rodenburg, R. J., van der Knaap, M. S., Willemsen, M. A., Sperl, W., Laugel, V., Ostergaard, E., Tarnopolsky, M., Martin, M. A., Nesbitt, V., Fletcher, J., Edvardson, S., Procaccio, V., Slama, A., van den Heuvel, L. P., & Smeitink, J. A. (2012). Natural disease course and genotype-phenotype correlations in Complex I deficiency caused by nuclear gene defects: what we learned from 130 cases. Journal of inherited metabolic disease, 35(5), 737–747. https://doi.org/10.1007/s10545-012-9492-z

39. Smeitink, J., & van den Heuvel, L. (1999). Human mitochondrial complex I in health and disease. American journal of human genetics, 64(6), 1505–1510. https://doi.org/10.1086/302432

40. Lebon, S., Rodriguez, D., Bridoux, D., Zerrad, A., Rötig, A., Munnich, A., Legrand, A., & Slama, A. (2007). A novel mutation in the human complex I NDUFS7 subunit associated with Leigh syndrome. Molecular genetics and metabolism, 90(4), 379–382. https://doi.org/10.1016/j.ymgme.2006.12.007

41. Lebon, S., Minai, L., Chretien, D., Corcos, J., Serre, V., Kadhom, N., Steffann, J., Pauchard, J. Y., Munnich, A., Bonnefont, J. P., & Rötig, A. (2007). A novel mutation of the NDUFS7 gene leads to activation of a cryptic exon and impaired assembly of mitochondrial complex I in a patient with Leigh syndrome. Molecular genetics and metabolism, 92(1-2), 104–108. https://doi.org/10.1016/j.ymgme.2007.05.010

42. Loeffen, J., Smeitink, J., Triepels, R., Smeets, R., Schuelke, M., Sengers, R., Trijbels, F., Hamel, B., Mullaart, R., & van den Heuvel, L. (1998). The first nuclear-encoded complex I mutation in a patient with Leigh syndrome. American journal of human genetics, 63(6), 1598–1608. https://doi.org/10.1086/302154

43. Procaccio, V., & Wallace, D. C. (2004). Late-onset Leigh syndrome in a patient with mitochondrial complex I NDUFS8 mutations. Neurology, 62(10), 1899–1901. https://doi.org/10.1212/01.wnl.0000125251.56131.65

44. Shabannejadian, F., Masoomizadeh, S. Z., & Andashti, B. (2023). Molecular analysis of gene variants in an Iranian family with psychomotor retardation mitochondrial disorder patient. Clinical case reports, 11(5), e7308. https://doi.org/10.1002/ccr3.7308

45. Gowda, V. K., Bylappa, A. Y., Kinhal, U., Srinivasan, V. M., & Vamyanmane, D. K. (2023). Mitochondrial Complex I Deficiency Masquerading as Stroke-Like Episode Clinically and as Alexander Disease Radiologically Following Chicken Pox. Annals of Indian Academy of Neurology, 26(6), 977–979. https://doi.org/10.4103/aian.aian_339_23

46. Tuppen, H. A., Hogan, V. E., He, L., Blakely, E. L., Worgan, L., Al-Dosary, M., Saretzki, G., Alston, C. L., Morris, A. A., Clarke, M., Jones, S., Devlin, A. M., Mansour, S., Chrzanowska-Lightowlers, Z. M., Thorburn, D. R., McFarland, R., & Taylor, R. W. (2010). The p.M292T NDUFS2 mutation causes complex I-deficient Leigh syndrome in multiple families. Brain : a journal of neurology, 133(10), 2952–2963. https://doi.org/10.1093/brain/awq232

47. Andzelm, M. M., Balasubramaniam, S., Yang, E., Compton, A. G., Millington, K., Zhu, J., Anselm, I., Rodan, L. H., Thorburn, D. R., Christodoulou, J., & Srivastava, S. (2022). Expansion of the clinical and neuroimaging spectrum associated with NDUFS8-related disorder. JIMD reports, 63(5), 391–399. https://doi.org/10.1002/jmd2.12303

48. Schuelke, M., Smeitink, J., Mariman, E., Loeffen, J., Plecko, B., Trijbels, F., Stöckler-Ipsiroglu, S., & van den Heuvel, L. (1999). Mutant NDUFV1 subunit of mitochondrial complex I causes leukodystrophy and myoclonic epilepsy. Nature genetics, 21(3), 260–261. https://doi.org/10.1038/6772

49. Borna, N. N., Kishita, Y., Sakai, N., Hamada, Y., Kamagata, K., Kohda, M., Ohtake, A., Murayama, K., & Okazaki, Y. (2020). Leigh Syndrome Due to NDUFV1 Mutations Initially Presenting as LBSL. Genes, 11(11), 1325. https://doi.org/10.3390/genes11111325

50. Wadhwa, Y., Rohilla, S., & Kaushik, J. S. (2018). Cystic Leucoencephalopathy in NDUFV1 Mutation. Indian journal of pediatrics, 85(12), 1128–1131. https://doi.org/10.1007/s12098-018-2721-1

51. Srivastava, A., Srivastava, K. R., Hebbar, M., Galada, C., Kadavigrere, R., Su, F., Cao, X., Chinnaiyan, A. M., Girisha, K. M., Shukla, A., & Bielas, S. L. (2018). Genetic diversity of NDUFV1-dependent mitochondrial complex I deficiency. European journal of human genetics : EJHG, 26(11), 1582–1587. https://doi.org/10.1038/s41431-018-0209-0

52. Gschwind, M., Garcia Segarra, N., Schaller, A., Bolognini, R., Nuoffer, J. M., Hourez, R., Deprez, M., Lhermitte, B., Maeder, P., Tran, C., & Kuntzer, T. (2022). Early-onset leukoencephalomyelopathy due to a biallelic NDUFV1 variant in a mid-forties patient. Annals of clinical and translational neurology, 9(6), 888–892. https://doi.org/10.1002/acn3.51556

53. Becker, N., Sharma, A., Gosse, M., Kubat, B., & Conway, K. S. (2022). The neuropathologic findings in a case of progressive cavitating leukoencephalopathy due to NDUFV1 pathogenic variants. Acta neuropathologica communications, 10(1), 142. https://doi.org/10.1186/s40478-022-01445-1

54. Incecik, F., Herguner, O. M., Besen, S., Bozdoğan, S. T., & Mungan, N. O. (2018). Late-Onset Leigh Syndrome due to NDUFV1 Mutation in a 10-Year-Old Boy Initially Presenting with Ataxia. Journal of pediatric neurosciences, 13(2), 205–207. https://doi.org/10.4103/jpn.JPN_138_17

55. Lieber, D. S., Calvo, S. E., Shanahan, K., Slate, N. G., Liu, S., Hershman, S. G., Gold, N. B., Chapman, B. A., Thorburn, D. R., Berry, G. T., Schmahmann, J. D., Borowsky, M. L., Mueller, D. M., Sims, K. B., & Mootha, V. K. (2013). Targeted exome sequencing of suspected mitochondrial disorders. Neurology, 80(19), 1762–1770. https://doi.org/10.1212/WNL.0b013e3182918c40

56. Kiss, S., Christodoulou, J., Thorburn, D. R., Freeman, J. L., Kornberg, A. J., Mandelstam, S., Compton, A. G., Cummings, B., Pais, L., Yaplito-Lee, J., & White, S. M. (2023). A cryptic pathogenic NDUFV1 variant identified by RNA-seq in a patient with normal complex I activity in muscle and transient magnetic resonance imaging changes. American journal of medical genetics. Part A, 191(6), 1599–1606. https://doi.org/10.1002/ajmg.a.63170

57. Baertling, F., Sánchez-Caballero, L., van den Brand, M. A. M., Distelmaier, F., Janssen, M. C. H., Rodenburg, R. J. T., Smeitink, J. A. M., & Nijtmans, L. G. J. (2018). A Heterozygous NDUFV1 Variant Aggravates Mitochondrial Complex I Deficiency in a Family with a Homoplasmic ND1 Variant. The Journal of pediatrics, 196, 309–313.e3. https://doi.org/10.1016/j.jpeds.2017.12.043

58. Vilain, C., Rens, C., Aeby, A., Balériaux, D., Van Bogaert, P., Remiche, G., Smet, J., Van Coster, R., Abramowicz, M., & Pirson, I. (2012). A novel NDUFV1 gene mutation in complex I deficiency in consanguineous siblings with brainstem lesions and Leigh syndrome. Clinical genetics, 82(3), 264–270. https://doi.org/10.1111/j.1399-0004.2011.01743.x

59. Breningstall, G. N., Shoffner, J., & Patterson, R. J. (2008). Siblings with leukoencephalopathy. Seminars in pediatric neurology, 15(4), 212–215. https://doi.org/10.1016/j.spen.2008.10.013

60. Laugel, V., This-Bernd, V., Cormier-Daire, V., Speeg-Schatz, C., de Saint-Martin, A., & Fischbach, M. (2007). Early-onset ophthalmoplegia in Leigh-like syndrome due to NDUFV1 mutations. Pediatric neurology, 36(1), 54–57. https://doi.org/10.1016/j.pediatrneurol.2006.08.007

61. Marin, S. E., Mesterman, R., Robinson, B., Rodenburg, R. J., Smeitink, J., & Tarnopolsky, M. A. (2013). Leigh syndrome associated with mitochondrial complex I deficiency due to novel mutations In NDUFV1 and NDUFS2. Gene, 516(1), 162–167. https://doi.org/10.1016/j.gene.2012.12.024

62. Zafeiriou, D. I., Rodenburg, R. J., Scheffer, H., van den Heuvel, L. P., Pouwels, P. J., Ververi, A., Athanasiadou-Piperopoulou, F., & van der Knaap, M. S. (2008). MR spectroscopy and serial

magnetic resonance imaging in a patient with mitochondrial cystic leukoencephalopathy due to complex I deficiency and NDUFV1 mutations and mild clinical course. Neuropediatrics, 39(3), 172–175. https://doi.org/10.1055/s-0028-1093336

63. Cameron, J. M., MacKay, N., Feigenbaum, A., Tarnopolsky, M., Blaser, S., Robinson, B. H., & Schulze, A. (2015). Exome sequencing identifies complex I NDUFV2 mutations as a novel cause of Leigh syndrome. European journal of paediatric neurology : EJPN : official journal of the European Paediatric Neurology Society, 19(5), 525–532. https://doi.org/10.1016/j.ejpn.2015.05.002

64. Bénit, P., Beugnot, R., Chretien, D., Giurgea, I., De Lonlay-Debeney, P., Issartel, J. P., Corral-Debrinski, M., Kerscher, S., Rustin, P., Rötig, A., & Munnich, A. (2003). Mutant NDUFV2 subunit of mitochondrial complex I causes early onset hypertrophic cardiomyopathy and encephalopathy. Human mutation, 21(6), 582–586. https://doi.org/10.1002/humu.10225

65. Pagniez-Mammeri, H., Lombes, A., Brivet, M., Ogier-de Baulny, H., Landrieu, P., Legrand, A., & Slama, A. (2009). Rapid screening for nuclear genes mutations in isolated respiratory chain complex I defects. Molecular genetics and metabolism, 96(4), 196–200. https://doi.org/10.1016/j.ymgme.2008.12.003

66. Kishita, Y., Shimura, M., Kohda, M., Fushimi, T., Nitta, K. R., Yatsuka, Y., Hirose, S., Ideguchi, H., Ohtake, A., Murayama, K., & Okazaki, Y. (2021). Genome sequencing and RNA-seq analyses of mitochondrial complex I deficiency revealed Alu insertion-mediated deletion in NDUFV2. Human mutation, 42(11), 1422–1428. https://doi.org/10.1002/humu.24274

67. Fernandez-Moreira, D., Ugalde, C., Smeets, R., Rodenburg, R. J., Lopez-Laso, E., Ruiz-Falco, M. L., Briones, P., Martin, M. A., Smeitink, J. A., & Arenas, J. (2007). X-linked NDUFA1 gene mutations associated with mitochondrial encephalomyopathy. Annals of neurology, 61(1), 73–83. https://doi.org/10.1002/ana.21036

68. Miyauchi, A., Osaka, H., Nagashima, M., Kuwajima, M., Monden, Y., Kohda, M., Kishita, Y., Okazaki, Y., Murayama, K., Ohtake, A., & Yamagata, T. (2018). Leigh syndrome with spinal cord involvement due to a hemizygous NDUFA1 mutation. Brain & development, 40(6), 498–502. https://doi.org/10.1016/j.braindev.2018.02.007

69. Potluri, P., Davila, A., Ruiz-Pesini, E., Mishmar, D., O'Hearn, S., Hancock, S., Simon, M., Scheffler, I. E., Wallace, D. C., & Procaccio, V. (2009). A novel NDUFA1 mutation leads to a progressive mitochondrial complex I-specific neurodegenerative disease. Molecular genetics and metabolism, 96(4), 189–195. https://doi.org/10.1016/j.ymgme.2008.12.004

70. Mayr, J. A., Bodamer, O., Haack, T. B., Zimmermann, F. A., Madignier, F., Prokisch, H., Rauscher, C., Koch, J., & Sperl, W. (2011). Heterozygous mutation in the X chromosomal NDUFA1 gene in a girl with complex I deficiency. Molecular genetics and metabolism, 103(4), 358–361. https://doi.org/10.1016/j.ymgme.2011.04.010

71. Uehara, N., Mori, M., Tokuzawa, Y., Mizuno, Y., Tamaru, S., Kohda, M., Moriyama, Y., Nakachi, Y., Matoba, N., Sakai, T., Yamazaki, T., Harashima, H., Murayama, K., Hattori, K., Hayashi, J., Yamagata, T., Fujita, Y., Ito, M., Tanaka, M., Nibu, K., … Okazaki, Y. (2014). New MT-ND6 and NDUFA1 mutations in mitochondrial respiratory chain disorders. Annals of clinical and translational neurology, 1(5), 361–369. https://doi.org/10.1002/acn3.59

72. Bindu, P. S., Sonam, K., Chiplunkar, S., Govindaraj, P., Nagappa, M., Vekhande, C. C., Aravinda, H. R., Ponmalar, J. J., Mahadevan, A., Gayathri, N., Bharath, M. S., Sinha, S., & Taly, A. B. (2018). Mitochondrial leukoencephalopathies: A border zone between acquired and inherited white matter disorders in children?. Multiple sclerosis and related disorders, 20, 84–92. https://doi.org/10.1016/j.msard.2018.01.003

73. Hoefs, S. J., Dieteren, C. E., Distelmaier, F., Janssen, R. J., Epplen, A., Swarts, H. G., Forkink, M., Rodenburg, R. J., Nijtmans, L. G., Willems, P. H., Smeitink, J. A., & van den Heuvel, L. P. (2008). NDUFA2 complex I mutation leads to Leigh disease. American journal of human genetics, 82(6), 1306–1315. https://doi.org/10.1016/j.ajhg.2008.05.007

74. Perrier, S., Gauquelin, L., Tétreault, M., Tran, L. T., Webb, N., Srour, M., Mitchell, J. J., Brunel-Guitton, C., Majewski, J., Long, V., Keller, S., Gambello, M. J., Simons, C., Care4Rare Canada Consortium, Vanderver, A., & Bernard, G. (2018). Recessive mutations in NDUFA2 cause mitochondrial leukoencephalopathy. Clinical genetics, 93(2), 396–400. https://doi.org/10.1111/cge.13126

75. Alagia, M., Cappuccio, G., Torella, A., D'Amico, A., Mazio, F., Romano, A., Fecarotta, S., Casari, G., Nigro, V., TUDP, & Brunetti-Pierri, N. (2020). Cavitating and tigroid-like leukoencephalopathy in a case of NDUFA2-related disorder. JIMD reports, 52(1), 11–16. https://doi.org/10.1002/jmd2.12094

76. van den Bosch, B. J., Gerards, M., Sluiter, W., Stegmann, A. P., Jongen, E. L., Hellebrekers, D. M., Oegema, R., Lambrichs, E. H., Prokisch, H., Danhauser, K., Schoonderwoerd, K., de Coo, I. F., & Smeets, H. J. (2012). Defective NDUFA9 as a novel cause of neonatally fatal complex I disease. Journal of medical genetics, 49(1), 10–15. https://doi.org/10.1136/jmedgenet-2011-100466

77. Baertling, F., Sánchez-Caballero, L., van den Brand, M. A. M., Fung, C. W., Chan, S. H., Wong, V. C., Hellebrekers, D. M. E., de Coo, I. F. M., Smeitink, J. A. M., Rodenburg, R. J. T., & Nijtmans, L. G. J. (2018). NDUFA9 point mutations cause a variable mitochondrial complex I assembly defect. Clinical genetics, 93(1), 111–118. https://doi.org/10.1111/cge.13089

78. Singh, R., Padmanabha, H., Arunachal, G., Mailankody, P., & Mahale, R. R. (2023). Childhood-Onset Generalized Dystonia Due to NDUFA9 Gene Mutation: An Expansion of Mutations Causing Leigh's Syndrome. Annals of Indian Academy of Neurology, 26(4), 606–608. https://doi.org/10.4103/aian.aian_274_23

79. Nesti, C., Ticci, C., Rubegni, A., Doccini, S., Scaturro, G., Vetro, A., Guerrini, R., Santorelli, F. M., & Procopio, E. (2023). Additive effect of DNAJC30 and NDUFA9 mutations causing Leigh syndrome. Journal of neurology, 270(6), 3266–3269. https://doi.org/10.1007/s00415-023-11673-7

80. Hoefs, S. J., van Spronsen, F. J., Lenssen, E. W., Nijtmans, L. G., Rodenburg, R. J., Smeitink, J. A., & van den Heuvel, L. P. (2011). NDUFA10 mutations cause complex I deficiency in a patient with Leigh disease. European journal of human genetics : EJHG, 19(3), 270–274. https://doi.org/10.1038/ejhg.2010.204

81. Kohda, M., Tokuzawa, Y., Kishita, Y., Nyuzuki, H., Moriyama, Y., Mizuno, Y., Hirata, T., Yatsuka, Y., Yamashita-Sugahara, Y., Nakachi, Y., Kato, H., Okuda, A., Tamaru, S., Borna, N. N., Banshoya, K., Aigaki, T., Sato-Miyata, Y., Ohnuma, K., Suzuki, T., Nagao, A., … Okazaki, Y. (2016). A Comprehensive Genomic Analysis Reveals the Genetic Landscape of Mitochondrial Respiratory Chain Complex Deficiencies. PLoS genetics, 12(1), e1005679. https://doi.org/10.1371/journal.pgen.1005679

82. Minoia, F., Bertamino, M., Picco, P., Severino, M., Rossi, A., Fiorillo, C., Minetti, C., Nesti, C., Santorelli, F. M., & Di Rocco, M. (2017). Widening the Heterogeneity of Leigh Syndrome: Clinical, Biochemical, and Neuroradiologic Features in a Patient Harboring a NDUFA10 Mutation. JIMD reports, 37, 37–43. https://doi.org/10.1007/8904_2017_9

83. Duc, N. M., Thu, N. T. M., Bui, C. B., Hoa, G., & Le Trung Hieu, N. (2023). Genotype and phenotype characteristics of West syndrome in 20 Vietnamese children: Two novel variants detected

by next-generation sequencing. Epilepsy research, 190, 107094. https://doi.org/10.1016/j.eplepsyres.2023.107094

84. Yahya, V., Spagnolo, F., Di Maggio, G., Leopizzi, E., De Marco, P., Fortunato, F., Comi, G. P., Rini, A., Monfrini, E., & Di Fonzo, A. (2022). Juvenile-onset dystonia with spasticity in Leigh syndrome caused by a novel NDUFA10 variant. Parkinsonism & related disorders, 104, 85–87. https://doi.org/10.1016/j.parkreldis.2022.10.016

85. Peverelli, L., Legati, A., Lamantea, E., Nasca, A., Lerario, A., Galimberti, V., Ghezzi, D., & Lamperti, C. (2019). New missense variants of NDUFA11 associated with late-onset myopathy. Muscle & nerve, 60(2), E11–E14. https://doi.org/10.1002/mus.26511

86. Berger, I., Hershkovitz, E., Shaag, A., Edvardson, S., Saada, A., & Elpeleg, O. (2008). Mitochondrial complex I deficiency caused by a deleterious NDUFA11 mutation. Annals of neurology, 63(3), 405–408. https://doi.org/10.1002/ana.21332

87. Torraco, A., Nasca, A., Verrigni, D., Pennisi, A., Zaki, M. S., Olivieri, G., Assouline, Z., Martinelli, D., Maroofian, R., Rizza, T., Di Nottia, M., Invernizzi, F., Lamantea, E., Longo, D., Houlden, H., Prokisch, H., Rötig, A., Dionisi-Vici, C., Bertini, E., Ghezzi, D., … Diodato, D. (2021). Novel NDUFA12 variants are associated with isolated complex I defect and variable clinical manifestation. Human mutation, 42(6), 699–710. https://doi.org/10.1002/humu.24195

88. Ostergaard, E., Rodenburg, R. J., van den Brand, M., Thomsen, L. L., Duno, M., Batbayli, M., Wibrand, F., & Nijtmans, L. (2011). Respiratory chain complex I deficiency due to NDUFA12 mutations as a new cause of Leigh syndrome. Journal of medical genetics, 48(11), 737–740. https://doi.org/10.1136/jmg.2011.088856

89. Speer, R. R., Ezeanya, U. C., Beaudoin, S. J., Glass, K. M., & Oji-Mmuo, C. N. (2020). Term Neonate Presenting with the Combined Occurrence of Mucolipidosis Type II and Leigh Syndrome. Journal of pediatric genetics, 9(2), 137–141. https://doi.org/10.1055/s-0039-1700519

90. Magrinelli, F., Cali, E., Braga, V. L., Yis, U., Tomoum, H., Shamseldin, H., Raiman, J., Kernstock, C., Rezende Filho, F. M., Barsottini, O. G. P., Taylor, R. W., Østergaard, E., Tamim, A., Schäferhoff, K., Sallum, J. M. F., Zaki, M. S., Kok, F., Bhatia, K. P., Wissinger, B., Sergeant, K., … Maroofian, R. (2022). Biallelic Loss-of-Function NDUFA12 Variants Cause a Wide Phenotypic Spectrum from Leigh/Leigh-Like Syndrome to Isolated Optic Atrophy. Movement disorders clinical practice, 9(2), 218–228. https://doi.org/10.1002/mdc3.13398

91. Angebault, C., Charif, M., Guegen, N., Piro-Megy, C., Mousson de Camaret, B., Procaccio, V., Guichet, P. O., Hebrard, M., Manes, G., Leboucq, N., Rivier, F., Hamel, C. P., Lenaers, G., & Roubertie, A. (2015). Mutation in NDUFA13/GRIM19 leads to early onset hypotonia, dyskinesia and sensorial deficiencies, and mitochondrial complex I instability. Human molecular genetics, 24(14), 3948–3955. https://doi.org/10.1093/hmg/ddv133

92. González-Quintana, A., García-Consuegra, I., Belanger-Quintana, A., Serrano-Lorenzo, P., Lucia, A., Blázquez, A., Docampo, J., Ugalde, C., Morán, M., Arenas, J., & Martín, M. A. (2020). Novel NDUFA13 Mutations Associated with OXPHOS Deficiency and Leigh Syndrome: A Second Family Report. Genes, 11(8), 855. https://doi.org/10.3390/genes11080855

93. Dunning, C. J., McKenzie, M., Sugiana, C., Lazarou, M., Silke, J., Connelly, A., Fletcher, J. M., Kirby, D. M., Thorburn, D. R., & Ryan, M. T. (2007). Human CIA30 is involved in the early assembly of mitochondrial complex I and mutations in its gene cause disease. The EMBO journal, 26(13), 3227–3237. https://doi.org/10.1038/sj.emboj.7601748

94. Wu, L., Liao, X., Yang, S., & Gan, S. (2021). Krabbe Disease Associated With Mitochondrial Dysfunction in a Chinese Family. Frontiers in neurology, 12, 750095. https://doi.org/10.3389/fneur.2021.750095

95. Wu, L., Peng, J., Ma, Y., He, F., Deng, X., Wang, G., Lifen, Y., & Yin, F. (2016). Leukodystrophy associated with mitochondrial complex I deficiency due to a novel mutation in the NDUFAF1 gene. Mitochondrial DNA. Part A, DNA mapping, sequencing, and analysis, 27(2), 1034–1037. https://doi.org/10.3109/19401736.2014.926543

96. Fassone, E., Taanman, J. W., Hargreaves, I. P., Sebire, N. J., Cleary, M. A., Burch, M., & Rahman, S. (2011). Mutations in the mitochondrial complex I assembly factor NDUFAF1 cause fatal infantile hypertrophic cardiomyopathy. Journal of medical genetics, 48(10), 691–697. https://doi.org/10.1136/jmedgenet-2011-100340

97. Nouws, J., Nijtmans, L., Houten, S. M., van den Brand, M., Huynen, M., Venselaar, H., Hoefs, S., Gloerich, J., Kronick, J., Hutchin, T., Willems, P., Rodenburg, R., Wanders, R., van den Heuvel, L., Smeitink, J., & Vogel, R. O. (2010). Acyl-CoA dehydrogenase 9 is required for the biogenesis of oxidative phosphorylation complex I. Cell metabolism, 12(3), 283–294. https://doi.org/10.1016/j.cmet.2010.08.002

98. Abu Hanna, F., Zehavi, Y., Cohen-Barak, E., Khayat, M., Warwar, N., Shreter, R., Rodenburg, R. J., & Spiegel, R. (2024). Lack of mitochondrial complex I assembly factor NDUFAF2 results in a distinctive infantile-onset brainstem neurodegenerative disease with early lethality. Orphanet journal of rare diseases, 19(1), 92. https://doi.org/10.1186/s13023-024-03094-0

99. Sabharwal, A., Gupta, V., Kv, S., Kumar Manokaran, R., Verma, A., Mishra, A., Bhoyar, R. C., Jain, A., Sivadas, A., Rawat, S., Jolly, B., Mohanty, S., Gulati, S., Gupta, N., Kabra, M., Scaria, V., & Sivasubbu, S. (2024). Whole genome sequencing followed by functional analysis of genomic deletion encompassing ERCC8 and NDUFAF2 genes in a non-consanguineous Indian family reveals dysfunctional mitochondrial bioenergetics leading to infant mortality. Mitochondrion, 75, 101844. https://doi.org/10.1016/j.mito.2024.101844

100. Marshall, A. E., Brady, L., Yeh, E., Mears, A. J., Lacaria, M., Chakraborty, P., Tarnopolsky, M. A., & Kernohan, K. D. (2024). Next generation sequencing reveals novel compound heterozygous deletions in NDUFAF2 in a child with mitochondrial complex I deficiency, nuclear type 10. American journal of medical genetics. Part A, 194(7), e63590. https://doi.org/10.1002/ajmg.a.63590

101. Herzer, M., Koch, J., Prokisch, H., Rodenburg, R., Rauscher, C., Radauer, W., Forstner, R., Pilz, P., Rolinski, B., Freisinger, P., Mayr, J. A., & Sperl, W. (2010). Leigh disease with brainstem involvement in complex I deficiency due to assembly factor NDUFAF2 defect. Neuropediatrics, 41(1), 30–34. https://doi.org/10.1055/s-0030-1255062

102. Janssen, R. J., Distelmaier, F., Smeets, R., Wijnhoven, T., Østergaard, E., Jaspers, N. G., Raams, A., Kemp, S., Rodenburg, R. J., Willems, P. H., van den Heuvel, L. P., Smeitink, J. A., & Nijtmans, L. G. (2009). Contiguous gene deletion of ELOVL7, ERCC8 and NDUFAF2 in a patient with a fatal multisystem disorder. Human molecular genetics, 18(18), 3365–3374. https://doi.org/10.1093/hmg/ddp276

103. Ghaloul-Gonzalez, L., Goldstein, A., Walsh Vockley, C., Dobrowolski, S. F., Biery, A., Irani, A., Ibarra, J., Morton, D. H., Mohsen, A. W., & Vockley, J. (2016). Mitochondrial respiratory chain disorders in the Old Order Amish population. Molecular genetics and metabolism, 118(4), 296–303. https://doi.org/10.1016/j.ymgme.2016.06.005

104. Saada, A., Vogel, R. O., Hoefs, S. J., van den Brand, M. A., Wessels, H. J., Willems, P. H., Venselaar, H., Shaag, A., Barghuti, F., Reish, O., Shohat, M., Huynen, M. A., Smeitink, J. A., van den

Heuvel, L. P., & Nijtmans, L. G. (2009). Mutations in NDUFAF3 (C3ORF60), encoding an NDUFAF4 (C6ORF66)-interacting complex I assembly protein, cause fatal neonatal mitochondrial disease. American journal of human genetics, 84(6), 718–727. https://doi.org/10.1016/j.ajhg.2009.04.020

105. Baertling, F., Sánchez-Caballero, L., Timal, S., van den Brand, M. A., Ngu, L. H., Distelmaier, F., Rodenburg, R. J., & Nijtmans, L. G. (2017). Mutations in mitochondrial complex I assembly factor NDUFAF3 cause Leigh syndrome. Molecular genetics and metabolism, 120(3), 243–246. https://doi.org/10.1016/j.ymgme.2016.12.005

106. Ishiyama, A., Muramatsu, K., Uchino, S., Sakai, C., Matsushima, Y., Makioka, N., Ogata, T., Suzuki, E., Komaki, H., Sasaki, M., Mimaki, M., Goto, Y. I., & Nishino, I. (2018). NDUFAF3 variants that disrupt mitochondrial complex I assembly may associate with cavitating leukoencephalopathy. Clinical genetics, 93(5), 1103–1106. https://doi.org/10.1111/cge.13215

107. van der Ven, A. T., Cabrera-Orefice, A., Wente, I., Feichtinger, R. G., Tsiakas, K., Weiss, D., Bierhals, T., Scholle, L., Prokisch, H., Kopajtich, R., Santer, R., Mayr, J. A., Hempel, M., & Wittig, I. (2023). Expanding the phenotypic and biochemical spectrum of NDUFAF3-related mitochondrial disease. Molecular genetics and metabolism, 140(3), 107675. https://doi.org/10.1016/j.ymgme.2023.107675

108. Baertling, F., Sánchez-Caballero, L., van den Brand, M. A. M., Wintjes, L. T., Brink, M., van den Brandt, F. A., Wilson, C., Rodenburg, R. J. T., & Nijtmans, L. G. J. (2017). NDUFAF4 variants are associated with Leigh syndrome and cause a specific mitochondrial complex I assembly defect. European journal of human genetics : EJHG, 25(11), 1273–1277. https://doi.org/10.1038/ejhg.2017.133

109. Ugarteburu, O., Teresa Garcia-Silva, M., Aldamiz-Echevarria, L., Gort, L., Garcia-Villoria, J., Tort, F., & Ribes, A. (2020). Complex I deficiency, due to NDUFAF4 mutations, causes severe mitochondrial dysfunction and is associated to early death and dysmorphia. Mitochondrion, 55, 78–84. https://doi.org/10.1016/j.mito.2020.09.003

110. Simon, M. T., Eftekharian, S. S., Stover, A. E., Osborne, A. F., Braffman, B. H., Chang, R. C., Wang, R. Y., Steenari, M. R., Tang, S., Hwu, P. W., Taft, R. J., Benke, P. J., & Abdenur, J. E. (2019). Novel mutations in the mitochondrial complex I assembly gene NDUFAF5 reveal heterogeneous phenotypes. Molecular genetics and metabolism, 126(1), 53–63. https://doi.org/10.1016/j.ymgme.2018.11.001

111. Chen, P. S., Lee, N. C., Sung, C. J., Liu, Y. W., Weng, W. C., Fan, P. C., Lee, W. T., Chien, Y. H., Wu, C. S., Sung, Y. F., Tsai, M. C., Lee, Y. C., Hsueh, H. W., Fan, S. M., Wu, M. C., Li, H., Chen, H. Y., Lin, H. I., Ou-Yang, C. H., Hwuh, W. L., … Lin, C. H. (2023). Phenotypic Heterogeneity in Patients with Mutations in the Mitochondrial Complex I Assembly Gene NDUFAF5. Movement disorders : official journal of the Movement Disorder Society, 38(12), 2217–2229. https://doi.org/10.1002/mds.29604

112. Bi, H., Guo, H., Wang, Q., Zhang, X., Zhao, Y., Li, J., Zhao, W., Tuo, H., & Zhang, Y. (2021). A Novel Variation in the Mitochondrial Complex I Assembly Factor NDUFAF5 Causes Isolated Bilateral Striatal Necrosis in Childhood. Frontiers in neurology, 12, 675616. https://doi.org/10.3389/fneur.2021.675616

113. Tong, W., Wang, Y., Lu, Y., Ye, T., Song, C., Xu, Y., Li, M., Ding, J., Duan, Y., Zhang, L., Gu, W., Zhao, X., Yang, X. A., & Jin, D. (2018). Whole-exome Sequencing Helps the Diagnosis and Treatment in Children with Neurodevelopmental Delay Accompanied Unexplained Dyspnea. Scientific reports, 8(1), 5214. https://doi.org/10.1038/s41598-018-23503-2

114. Wen, Y., Lu, G., Qiao, L., & Li, Y. (2022). A Leigh syndrome caused by compound heterozygous mutations on NDUFAF5 induce early infant death: A case report. Molecular genetics & genomic medicine, 10(1), e1852. https://doi.org/10.1002/mgg3.1852

115. Gouiza, I., Hechmi, M., Zioudi, A., Dallali, H., Kheriji, N., Charif, M., Le Mao, M., Galai, S., Kraoua, L., Ben Youssef-Turki, I., Kraoua, I., Lenaers, G., & Kefi, R. (2024). Expanding the genetic spectrum of mitochondrial diseases in Tunisia: novel variants revealed by whole-exome sequencing. Frontiers in genetics, 14, 1259826. https://doi.org/10.3389/fgene.2023.1259826

116. Sugiana, C., Pagliarini, D. J., McKenzie, M., Kirby, D. M., Salemi, R., Abu-Amero, K. K., Dahl, H. H., Hutchison, W. M., Vascotto, K. A., Smith, S. M., Newbold, R. F., Christodoulou, J., Calvo, S., Mootha, V. K., Ryan, M. T., & Thorburn, D. R. (2008). Mutation of C20orf7 disrupts complex I assembly and causes lethal neonatal mitochondrial disease. American journal of human genetics, 83(4), 468–478. https://doi.org/10.1016/j.ajhg.2008.09.009

117. Gerards, M., Sluiter, W., van den Bosch, B. J., de Wit, L. E., Calis, C. M., Frentzen, M., Akbari, H., Schoonderwoerd, K., Scholte, H. R., Jongbloed, R. J., Hendrickx, A. T., de Coo, I. F., & Smeets, H. J. (2010). Defective complex I assembly due to C20orf7 mutations as a new cause of Leigh syndrome. Journal of medical genetics, 47(8), 507–512. https://doi.org/10.1136/jmg.2009.067553

118. Saada, A., Edvardson, S., Shaag, A., Chung, W. K., Segel, R., Miller, C., Jalas, C., & Elpeleg, O. (2012). Combined OXPHOS complex I and IV defect, due to mutated complex I assembly factor C20ORF7. Journal of inherited metabolic disease, 35(1), 125–131. https://doi.org/10.1007/s10545-011-9348-y

119. Theunissen, T. E. J., Gerards, M., Hellebrekers, D. M. E. I., van Tienen, F. H., Kamps, R., Sallevelt, S. C. E. H., Hartog, E. N. M. M., Scholte, H. R., Verdijk, R. M., Schoonderwoerd, K., de Coo, I. F. M., Szklarczyk, R., & Smeets, H. J. M. (2017). Selection and Characterization of Palmitic Acid Responsive Patients with an OXPHOS Complex I Defect. Frontiers in molecular neuroscience, 10, 336. https://doi.org/10.3389/fnmol.2017.00336

120. Zhang, J., Liu, M., Zhang, Z., Zhou, L., Kong, W., Jiang, Y., Wang, J., Xiao, J., & Wu, Y. (2019). Genotypic Spectrum and Natural History of Cavitating Leukoencephalopathies in Childhood. Pediatric neurology, 94, 38–47. https://doi.org/10.1016/j.pediatrneurol.2019.01.002

121. Hu, C., Li, X., Zhao, L., Shi, Y., Zhou, S., Wu, B., & Wang, Y. (2020). Clinical and molecular characterization of pediatric mitochondrial disorders in south of China. European journal of medical genetics, 63(8), 103898. https://doi.org/10.1016/j.ejmg.2020.103898

122. Mansukhani, S. A., Mehta, D. G., Renaud, D. L., Whealy, M. A., Chen, J. J., & Bhatti, M. T. (2021). Nuclear DNA Mutation Causing a Phenotypic Leber Hereditary Optic Neuropathy Plus. Ophthalmology, 128(4), 628–631. https://doi.org/10.1016/j.ophtha.2020.09.011

123. Legro, N. R., Kumar, A., & Aliu, E. (2022). Case report of atypical Leigh syndrome in an adolescent male with novel biallelic variants in NDUFAF5 and review of the natural history of NDUFAF5-related disorders. American journal of medical genetics. Part A, 188(3), 896–899. https://doi.org/10.1002/ajmg.a.62568

124. Kim, J., Lee, J., & Jang, D. H. (2022). NDUFAF6-Related Leigh Syndrome Caused by Rare Pathogenic Variants: A Case Report and the Focused Review of Literature. Frontiers in pediatrics, 10, 812408. https://doi.org/10.3389/fped.2022.812408

125. Baide-Mairena, H., Gaudó, P., Marti-Sánchez, L., Emperador, S., Sánchez-Montanez, A., Alonso-Luengo, O., Correa, M., Grau, A. M., Ortigoza-Escobar, J. D., Artuch, R., Vázquez, E., Del

Toro, M., Garrido-Pérez, N., Ruiz-Pesini, E., Montoya, J., Bayona-Bafaluy, M. P., & Pérez-Dueñas, B. (2019). Mutations in the mitochondrial complex I assembly factor NDUFAF6 cause isolated bilateral striatal necrosis and progressive dystonia in childhood. Molecular genetics and metabolism, 126(3), 250–258. https://doi.org/10.1016/j.ymgme.2019.01.001

126. Martikainen, M. H., Ng, Y. S., Gorman, G. S., Alston, C. L., Blakely, E. L., Schaefer, A. M., Chinnery, P. F., Burn, D. J., Taylor, R. W., McFarland, R., & Turnbull, D. M. (2016). Clinical, Genetic, and Radiological Features of Extrapyramidal Movement Disorders in Mitochondrial Disease. JAMA neurology, 73(6), 668–674. https://doi.org/10.1001/jamaneurol.2016.0355

127. Johnstone, T., Wang, J., Ross, D., Balanda, N., Huang, Y., Godfrey, R., Groden, C., Barton, B. R., Gahl, W., Toro, C., & Malicdan, M. C. V. (2020). Biallelic variants in two complex I genes cause abnormal splicing defects in probands with mild Leigh syndrome. Molecular genetics and metabolism, 131(1-2), 98–106. https://doi.org/10.1016/j.ymgme.2020.09.008

128. Gedikbasi, A., Toksoy, G., Karaca, M., Gulec, C., Balci, M. C., Gunes, D., Gunes, S., Aslanger, A. D., Unverengil, G., Karaman, B., Basaran, S., Demirkol, M., Gokcay, G. F., & Uyguner, Z. O. (2023). Clinical and bi-genomic DNA findings of patients suspected to have mitochondrial diseases. Frontiers in genetics, 14, 1191159. https://doi.org/10.3389/fgene.2023.1191159

129. Calvo, S. E., Compton, A. G., Hershman, S. G., Lim, S. C., Lieber, D. S., Tucker, E. J., Laskowski, A., Garone, C., Liu, S., Jaffe, D. B., Christodoulou, J., Fletcher, J. M., Bruno, D. L., Goldblatt, J., Dimauro, S., Thorburn, D. R., & Mootha, V. K. (2012). Molecular diagnosis of infantile mitochondrial disease with targeted next-generation sequencing. Science translational medicine, 4(118), 118ra10. https://doi.org/10.1126/scitranslmed.3003310

130. Alston, C. L., Howard, C., Oláhová, M., Hardy, S. A., He, L., Murray, P. G., O'Sullivan, S., Doherty, G., Shield, J. P., Hargreaves, I. P., Monavari, A. A., Knerr, I., McCarthy, P., Morris, A. A., Thorburn, D. R., Prokisch, H., Clayton, P. E., McFarland, R., Hughes, J., Crushell, E., … Taylor, R. W. (2016). A recurrent mitochondrial p.Trp22Arg NDUFB3 variant causes a distinctive facial appearance, short stature and a mild biochemical and clinical phenotype. Journal of medical genetics, 53(9), 634–641. https://doi.org/10.1136/jmedgenet-2015-103576

131. Haack, T. B., Madignier, F., Herzer, M., Lamantea, E., Danhauser, K., Invernizzi, F., Koch, J., Freitag, M., Drost, R., Hillier, I., Haberberger, B., Mayr, J. A., Ahting, U., Tiranti, V., Rötig, A., Iuso, A., Horvath, R., Tesarova, M., Baric, I., Uziel, G., … Prokisch, H. (2012). Mutation screening of 75 candidate genes in 152 complex I deficiency cases identifies pathogenic variants in 16 genes including NDUFB9. Journal of medical genetics, 49(2), 83–89. https://doi.org/10.1136/jmedgenet-2011-100577

132. Friederich, M. W., Erdogan, A. J., Coughlin, C. R., 2nd, Elos, M. T., Jiang, H., O'Rourke, C. P., Lovell, M. A., Wartchow, E., Gowan, K., Chatfield, K. C., Chick, W. S., Spector, E. B., Van Hove, J. L. K., & Riemer, J. (2017). Mutations in the accessory subunit NDUFB10 result in isolated complex I deficiency and illustrate the critical role of intermembrane space import for complex I holoenzyme assembly. Human molecular genetics, 26(4), 702–716. https://doi.org/10.1093/hmg/ddw431

133. Reinson, K., Kovacs-Nagy, R., Õiglane-Shlik, E., Pajusalu, S., Nõukas, M., Wintjes, L. T., van den Brandt, F. C. A., Brink, M., Acker, T., Ahting, U., Hahn, A., Schänzer, A., Haack, T. B., Rodenburg, R. J., & Õunap, K. (2019). Diverse phenotype in patients with complex I deficiency due to mutations in NDUFB11. European journal of medical genetics, 62(11), 103572. https://doi.org/10.1016/j.ejmg.2018.11.006

134. Shehata, B. M., Cundiff, C. A., Lee, K., Sabharwal, A., Lalwani, M. K., Davis, A. K., Agrawal, V., Sivasubbu, S., Iannucci, G. J., & Gibson, G. (2015). Exome sequencing of patients with histiocytoid cardiomyopathy reveals a de novo NDUFB11 mutation that plays a role in the pathogenesis of histiocytoid cardiomyopathy. American journal of medical genetics. Part A, 167A(9), 2114–2121. https://doi.org/10.1002/ajmg.a.37138

135. van Rahden, V. A., Fernandez-Vizarra, E., Alawi, M., Brand, K., Fellmann, F., Horn, D., Zeviani, M., & Kutsche, K. (2015). Mutations in NDUFB11, encoding a complex I component of the mitochondrial respiratory chain, cause microphthalmia with linear skin defects syndrome. American journal of human genetics, 96(4), 640–650. https://doi.org/10.1016/j.ajhg.2015.02.002

136. Rea, G., Homfray, T., Till, J., Roses-Noguer, F., Buchan, R. J., Wilkinson, S., Wilk, A., Walsh, R., John, S., McKee, S., Stewart, F. J., Murday, V., Taylor, R. W., Ashworth, M., Baksi, A. J., Daubeney, P., Prasad, S., Barton, P. J. R., Cook, S. A., & Ware, J. S. (2017). Histiocytoid cardiomyopathy and microphthalmia with linear skin defects syndrome: phenotypes linked by truncating variants in NDUFB11. Cold Spring Harbor molecular case studies, 3(1), a001271. https://doi.org/10.1101/mcs.a001271

137. Torraco, A., Bianchi, M., Verrigni, D., Gelmetti, V., Riley, L., Niceta, M., Martinelli, D., Montanari, A., Guo, Y., Rizza, T., Diodato, D., Di Nottia, M., Lucarelli, B., Sorrentino, F., Piemonte, F., Francisci, S., Tartaglia, M., Valente, E. M., Dionisi-Vici, C., Christodoulou, J., … Carrozzo, R. (2017). A novel mutation in NDUFB11 unveils a new clinical phenotype associated with lactic acidosis and sideroblastic anemia. Clinical genetics, 91(3), 441–447. https://doi.org/10.1111/cge.12790

138. Lichtenstein, D. A., Crispin, A. W., Sendamarai, A. K., Campagna, D. R., Schmitz-Abe, K., Sousa, C. M., Kafina, M. D., Schmidt, P. J., Niemeyer, C. M., Porter, J., May, A., Patnaik, M. M., Heeney, M. M., Kimmelman, A., Bottomley, S. S., Paw, B. H., Markianos, K., & Fleming, M. D. (2016). A recurring mutation in the respiratory complex 1 protein NDUFB11 is responsible for a novel form of X-linked sideroblastic anemia. Blood, 128(15), 1913–1917. https://doi.org/10.1182/blood-2016-05-719062

139. Puusepp, S., Reinson, K., Pajusalu, S., Murumets, Ü., Õiglane-Shlik, E., Rein, R., Talvik, I., Rodenburg, R. J., & Õunap, K. (2018). Effectiveness of whole exome sequencing in unsolved patients with a clinical suspicion of a mitochondrial disorder in Estonia. Molecular genetics and metabolism reports, 15, 80–89. https://doi.org/10.1016/j.ymgmr.2018.03.004

140. Amate-García, G., Ballesta-Martínez, M. J., Serrano-Lorenzo, P., Garrido-Moraga, R., González-Quintana, A., Blázquez, A., Rubio, J. C., García-Consuegra, I., Arenas, J., Ugalde, C., Morán, M., Guillén-Navarro, E., & Martín, M. A. (2023). A Novel Mutation Associated with Neonatal Lethal Cardiomyopathy Leads to an Alternative Transcript Expression in the X-Linked Complex I NDUFB11 Gene. International journal of molecular sciences, 24(2), 1743. https://doi.org/10.3390/ijms24021743

141. Alahmad, A., Nasca, A., Heidler, J., Thompson, K., Oláhová, M., Legati, A., Lamantea, E., Meisterknecht, J., Spagnolo, M., He, L., Alameer, S., Hakami, F., Almehdar, A., Ardissone, A., Alston, C. L., McFarland, R., Wittig, I., Ghezzi, D., & Taylor, R. W. (2020). Bi-allelic pathogenic variants in NDUFC2 cause early-onset Leigh syndrome and stalled biogenesis of complex I. EMBO molecular medicine, 12(11), e12619. https://doi.org/10.15252/emmm.202012619

142. 10.3389/fneur.2023.1292320

Other CI-associated genes:

1. Nouws, J., Nijtmans, L. G., Smeitink, J. A., & Vogel, R. O. (2012). Assembly factors as a new class of disease genes for mitochondrial complex I deficiency: cause, pathology and treatment options. Brain : a journal of neurology, 135(Pt 1), 12–22. https://doi.org/10.1093/brain/awr261

2. Gerards, M., Sluiter, W., van den Bosch, B. J., de Wit, L. E., Calis, C. M., Frentzen, M., Akbari, H., Schoonderwoerd, K., Scholte, H. R., Jongbloed, R. J., Hendrickx, A. T., de Coo, I. F., & Smeets, H. J. (2010). Defective complex I assembly due to C20orf7 mutations as a new cause of Leigh syndrome. Journal of medical genetics, 47(8), 507–512. https://doi.org/10.1136/jmg.2009.067553

3. Saada, A., Edvardson, S., Shaag, A., Chung, W. K., Segel, R., Miller, C., Jalas, C., & Elpeleg, O. (2012). Combined OXPHOS complex I and IV defect, due to mutated complex I assembly factor C20ORF7. Journal of inherited metabolic disease, 35(1), 125–131. https://doi.org/10.1007/s10545-011-9348-y

4. Sugiana, C., Pagliarini, D. J., McKenzie, M., Kirby, D. M., Salemi, R., Abu-Amero, K. K., Dahl, H. H., Hutchison, W. M., Vascotto, K. A., Smith, S. M., Newbold, R. F., Christodoulou, J., Calvo, S., Mootha, V. K., Ryan, M. T., & Thorburn, D. R. (2008). Mutation of C20orf7 disrupts complex I assembly and causes lethal neonatal mitochondrial disease. American journal of human genetics, 83(4), 468–478. https://doi.org/10.1016/j.ajhg.2008.09.009

5. Johnstone, T., Wang, J., Ross, D., Balanda, N., Huang, Y., Godfrey, R., Groden, C., Barton, B. R., Gahl, W., Toro, C., & Malicdan, M. C. V. (2020). Biallelic variants in two complex I genes cause abnormal splicing defects in probands with mild Leigh syndrome. Molecular genetics and metabolism, 131(1-2), 98–106. https://doi.org/10.1016/j.ymgme.2020.09.008

6. Friederich, M. W., Perez, F. A., Knight, K. M., Van Hove, R. A., Yang, S. P., Saneto, R. P., & Van Hove, J. L. K. (2020). Pathogenic variants in NUBPL result in failure to assemble the matrix arm of complex I and cause a complex leukoencephalopathy with thalamic involvement. Molecular genetics and metabolism, 129(3), 236–242. https://doi.org/10.1016/j.ymgme.2019.12.013

7. Kevelam, S. H., Rodenburg, R. J., Wolf, N. I., Ferreira, P., Lunsing, R. J., Nijtmans, L. G., Mitchell, A., Arroyo, H. A., Rating, D., Vanderver, A., van Berkel, C. G., Abbink, T. E., Heutink, P., & van der Knaap, M. S. (2013). NUBPL mutations in patients with complex I deficiency and a distinct MRI pattern. Neurology, 80(17), 1577–1583. https://doi.org/10.1212/WNL.0b013e31828f1914

8. Balint, B., Charlesworth, G., Stamelou, M., Carr, L., Mencacci, N. E., Wood, N. W., & Bhatia, K. P. (2019). Mitochondrial complex I NUBPL mutations cause combined dystonia with bilateral striatal necrosis and cerebellar atrophy. European journal of neurology, 26(9), 1240–1243. https://doi.org/10.1111/ene.13956

9. Tenisch, E. V., Lebre, A. S., Grévent, D., de Lonlay, P., Rio, M., Zilbovicius, M., Funalot, B., Desguerre, I., Brunelle, F., Rötig, A., Munnich, A., & Boddaert, N. (2012). Massive and exclusive pontocerebellar damage in mitochondrial disease and NUBPL mutations. Neurology, 79(4), 391. https://doi.org/10.1212/WNL.0b013e3182611232

10. Protasoni, M., Bruno, C., Donati, M. A., Mohamoud, K., Severino, M., Allegri, A., Robinson, A. J., Reyes, A., Zeviani, M., & Garone, C. (2020). Novel compound heterozygous pathogenic variants in nucleotide-binding protein like protein (NUBPL) cause leukoencephalopathy with multi-systemic involvement. Molecular genetics and metabolism, 129(1), 26–34. https://doi.org/10.1016/j.ymgme.2019.11.003Peter S, B., & Vandana G, S. (2022). Leukodystrophy Associated with Mitochondrial Complex 1 Deficiency Due to Mutation in NUBPL Gene-An Unusual

Follow-Up Finding. The Indian journal of radiology & imaging, 33(1), 132–135. https://doi.org/10.1055/s-0042-1758195

11. Kremer, L. S., Bader, D. M., Mertes, C., Kopajtich, R., Pichler, G., Iuso, A., Haack, T. B., Graf, E., Schwarzmayr, T., Terrile, C., Koňaříková, E., Repp, B., Kastenmüller, G., Adamski, J., Lichtner, P., Leonhardt, C., Funalot, B., Donati, A., Tiranti, V., Lombes, A., … Prokisch, H. (2017). Genetic diagnosis of Mendelian disorders via RNA sequencing. Nature communications, 8, 15824. https://doi.org/10.1038/ncomms15824

12. Boutaud, L., Ruzzenente, B., Tessier, A., Anselem, O., Pannier, E., Grotto, S., Talhi, N., Amram, D., Willems, M., Wells, C., Blanchet, P., Musizzano, Y., Jauny, C., Nitschke, P., Bole-Feysot, C., Bessières, B., Salhi, H., Achaiaa, A., Metodiev, M. D., Razavi, F., … Attié-Bitach, T. (2023). Neuropathological hallmarks of antenatal mitochondrial diseases with a corpus callosum defect. Brain : a journal of neurology, 146(5), 1804–1811. https://doi.org/10.1093/brain/awac417

13. Lake, N. J., Formosa, L. E., Stroud, D. A., Ryan, M. T., Calvo, S. E., Mootha, V. K., Morar, B., Procopis, P. G., Christodoulou, J., Compton, A. G., & Thorburn, D. R. (2019). A patient with homozygous nonsense variants in two Leigh syndrome disease genes: Distinguishing a dual diagnosis from a hypomorphic protein-truncating variant. Human mutation, 40(7), 893–898. https://doi.org/10.1002/humu.23753

14. Naber, M., Hellebrekers, D., Nievelstein, R. A. J., van Hasselt, P. M., van Jaarsveld, R. H., Cuppen, I., & Oegema, R. (2021). Deep intronic TIMMDC1 variant delays diagnosis of rapidly progressive complex I deficiency. European journal of medical genetics, 64(1), 104120. https://doi.org/10.1016/j.ejmg.2020.104120

15. Alston, C. L., Compton, A. G., Formosa, L. E., Strecker, V., Oláhová, M., Haack, T. B., Smet, J., Stouffs, K., Diakumis, P., Ciara, E., Cassiman, D., Romain, N., Yarham, J. W., He, L., De Paepe, B., Vanlander, A. V., Seneca, S., Feichtinger, R. G., Płoski, R., Rokicki, D., … Taylor, R. W. (2016). Biallelic Mutations in TMEM126B Cause Severe Complex I Deficiency with a Variable Clinical Phenotype. American journal of human genetics, 99(1), 217–227. https://doi.org/10.1016/j.ajhg.2016.05.021

16. Sánchez-Caballero, L., Ruzzenente, B., Bianchi, L., Assouline, Z., Barcia, G., Metodiev, M. D., Rio, M., Funalot, B., van den Brand, M. A., Guerrero-Castillo, S., Molenaar, J. P., Koolen, D., Brandt, U., Rodenburg, R. J., Nijtmans, L. G., & Rötig, A. (2016). Mutations in Complex I Assembly Factor TMEM126B Result in Muscle Weakness and Isolated Complex I Deficiency. American journal of human genetics, 99(1), 208–216. https://doi.org/10.1016/j.ajhg.2016.05.022

17. Pronicka, E., Piekutowska-Abramczuk, D., Ciara, E., Trubicka, J., Rokicki, D., Karkucińska-Więckowska, A., Pajdowska, M., Jurkiewicz, E., Halat, P., Kosińska, J., Pollak, A., Rydzanicz, M., Stawinski, P., Pronicki, M., Krajewska-Walasek, M., & Płoski, R. (2016). New perspective in diagnostics of mitochondrial disorders: two years' experience with whole-exome sequencing at a national paediatric centre. Journal of translational medicine, 14(1), 174. https://doi.org/10.1186/s12967-016-0930-9

18. Zhou, X., Lou, X., Zhou, Y., Xie, Y., Han, X., Dong, Q., Ying, X., Laurentinah, M. R., Zhang, L., Chen, Z., Li, D., Fang, H., Lyu, J., Yang, Y., & Wang, Y. (2023). Novel biallelic mutations in TMEM126B cause splicing defects and lead to Leigh-like syndrome with severe complex I deficiency. Journal of human genetics, 68(4), 239–246. https://doi.org/10.1038/s10038-022-01102-4

19. Theunissen, T. E. J., Gerards, M., Hellebrekers, D. M. E. I., van Tienen, F. H., Kamps, R., Sallevelt, S. C. E. H., Hartog, E. N. M. M., Scholte, H. R., Verdijk, R. M., Schoonderwoerd, K., de Coo, I. F. M., Szklarczyk, R., & Smeets, H. J. M. (2017). Selection and Characterization of Palmitic

Acid Responsive Patients with an OXPHOS Complex I Defect. Frontiers in molecular neuroscience, 10, 336. https://doi.org/10.3389/fnmol.2017.00336

20. Haack, T. B., Haberberger, B., Frisch, E. M., Wieland, T., Iuso, A., Gorza, M., Strecker, V., Graf, E., Mayr, J. A., Herberg, U., Hennermann, J. B., Klopstock, T., Kuhn, K. A., Ahting, U., Sperl, W., Wilichowski, E., Hoffmann, G. F., Tesarova, M., Hansikova, H., Zeman, J., … Prokisch, H. (2012). Molecular diagnosis in mitochondrial complex I deficiency using exome sequencing. Journal of medical genetics, 49(4), 277–283. https://doi.org/10.1136/jmedgenet-2012-100846

21. Set, K. K., & De Dios, K. (2022). Nonprogressive Mobile Dystonia in MTFMT-Related Mitochondrial Disease. Movement disorders clinical practice, 10(1), 145–147. https://doi.org/10.1002/mdc3.13595

22. Tucker, E. J., Hershman, S. G., Köhrer, C., Belcher-Timme, C. A., Patel, J., Goldberger, O. A., Christodoulou, J., Silberstein, J. M., McKenzie, M., Ryan, M. T., Compton, A. G., Jaffe, J. D., Carr, S. A., Calvo, S. E., RajBhandary, U. L., Thorburn, D. R., & Mootha, V. K. (2011). Mutations in MTFMT underlie a human disorder of formylation causing impaired mitochondrial translation. Cell metabolism, 14(3), 428–434. https://doi.org/10.1016/j.cmet.2011.07.010

23. La Piana, R., Weraarpachai, W., Ospina, L. H., Tetreault, M., Majewski, J., Care4Rare Canada Consortium, Bruce Pike, G., Decarie, J. C., Tampieri, D., Brais, B., & Shoubridge, E. A. (2017). Identification and functional characterization of a novel MTFMT mutation associated with selective vulnerability of the visual pathway and a mild neurological phenotype. Neurogenetics, 18(2), 97–103. https://doi.org/10.1007/s10048-016-0506-0

24. Pena, J. A., Lotze, T., Yang, Y., Umana, L., Walkiewicz, M., Hunter, J. V., & Scaglia, F. (2016). Methionyl-tRNA Formyltransferase (MTFMT) Deficiency Mimicking Acquired Demyelinating Disease. Journal of child neurology, 31(2), 215–219. https://doi.org/10.1177/0883073815587946

25. Hinttala, R., Sasarman, F., Nishimura, T., Antonicka, H., Brunel-Guitton, C., Schwartzentruber, J., Fahiminiya, S., Majewski, J., Faubert, D., Ostergaard, E., Smeitink, J. A., & Shoubridge, E. A. (2015). An N-terminal formyl methionine on COX 1 is required for the assembly of cytochrome c oxidase. Human molecular genetics, 24(14), 4103–4113. https://doi.org/10.1093/hmg/ddv149

26. Haack, T. B., Gorza, M., Danhauser, K., Mayr, J. A., Haberberger, B., Wieland, T., Kremer, L., Strecker, V., Graf, E., Memari, Y., Ahting, U., Kopajtich, R., Wortmann, S. B., Rodenburg, R. J., Kotzaeridou, U., Hoffmann, G. F., Sperl, W., Wittig, I., Wilichowski, E., Schottmann, G., … Freisinger, P. (2014). Phenotypic spectrum of eleven patients and five novel MTFMT mutations identified by exome sequencing and candidate gene screening. Molecular genetics and metabolism, 111(3), 342–352. https://doi.org/10.1016/j.ymgme.2013.12.010

27. Prasun, P., & Del Mar Pena, L. (2014). Late onset Leigh syndrome mimicking central nervous system vasculitis. Molecular genetics and metabolism reports, 1, 280–282. https://doi.org/10.1016/j.ymgmr.2014.07.004

28. Neeve, V. C., Pyle, A., Boczonadi, V., Gomez-Duran, A., Griffin, H., Santibanez-Koref, M., Gaiser, U., Bauer, P., Tzschach, A., Chinnery, P. F., & Horvath, R. (2013). Clinical and functional characterisation of the combined respiratory chain defect in two sisters due to autosomal recessive mutations in MTFMT. Mitochondrion, 13(6), 743–748. https://doi.org/10.1016/j.mito.2013.03.002

29. Hayhurst, H., de Coo, I. F. M., Piekutowska-Abramczuk, D., Alston, C. L., Sharma, S., Thompson, K., Rius, R., He, L., Hopton, S., Ploski, R., Ciara, E., Lake, N. J., Compton, A. G., Delatycki, M. B., Verrips, A., Bonnen, P. E., Jones, S. A., Morris, A. A., Shakespeare, D.,

Christodoulou, J., … Ng, Y. S. (2019). Leigh syndrome caused by mutations in MTFMT is associated with a better prognosis. Annals of clinical and translational neurology, 6(3), 515–524. https://doi.org/10.1002/acn3.725

30. Bai, R., Haude, K., Yang, E., Goldstein, A., & Anselm, I. (2020). First report of childhood progressive cerebellar atrophy due to compound heterozygous MTFMT variants. Clinical genetics, 97(5), 793–794. https://doi.org/10.1111/cge.13708

31. Hemelsoet, D. M., Vanlander, A. V., Smet, J., Vantroys, E., Acou, M., Goethals, I., Sante, T., Seneca, S., Menten, B., & Van Coster, R. (2018). Leigh syndrome followed by parkinsonism in an adult with homozygous c.626C>T mutation in MTFMT. Neurology. Genetics, 4(6), e298. https://doi.org/10.1212/NXG.0000000000000298

32. Bennett, J., Kerr, M., Greenway, S. C., Friederich, M. W., Van Hove, J. L. K., Hittel, D., & Khan, A. (2020). Improved lactate control with dichloroacetate in a case with severe neonatal lactic acidosis due to MTFMT mitochondrial translation disorder. Molecular genetics and metabolism reports, 24, 100616. https://doi.org/10.1016/j.ymgmr.2020.100616

33. Howard, C., Dev-Borman, A., Stokes, J., O'Rourke, D., Gillespie, C., Twomey, E., Knerr, I., & Boruah, R. (2022). Autonomic instability, arrhythmia and visual impairment in a new presentation of MTFMT-related mitochondrial disease. JIMD reports, 64(2), 150–155. https://doi.org/10.1002/jmd2.12355

34. Schwartz, M., & Vissing, J. (2002). Paternal inheritance of mitochondrial DNA. The New England journal of medicine, 347(8), 576–580. https://doi.org/10.1056/NEJMoa020350

35. Pulkes, T., Liolitsa, D., Wills, A. J., Hargreaves, I., Heales, S., & Hanna, M. G. (2005). Nonsense mutations in mitochondrial DNA associated with myalgia and exercise intolerance. Neurology, 64(6), 1091–1092. https://doi.org/10.1212/01.WNL.0000154471.33156.55

36. Hinttala, R., Smeets, R., Moilanen, J. S., Ugalde, C., Uusimaa, J., Smeitink, J. A., & Majamaa, K. (2006). Analysis of mitochondrial DNA sequences in patients with isolated or combined oxidative phosphorylation system deficiency. Journal of medical genetics, 43(11), 881–886. https://doi.org/10.1136/jmg.2006.042168

37. Vodopivec, I., Cho, T. A., Rizzo, J. F., 3rd, Frosch, M. P., & Sims, K. B. (2016). Mitochondrial Encephalopathy and Optic Neuropathy Due to m.10158 MT-ND3 Complex I Mutation Presenting in an Adult Patient: Case Report and Review of the Literature. The neurologist, 21(4), 61–65. https://doi.org/10.1097/NRL.0000000000000084

38. Borna, N. N., Kishita, Y., Shimura, M., Murayama, K., Ohtake, A., & Okazaki, Y. (2024). Identification of a novel MT-ND3 variant and restoring mitochondrial function by allotopic expression of MT-ND3 gene. Mitochondrion, 76, 101858. https://doi.org/10.1016/j.mito.2024.101858

39. Newstead, S. M., & Finsterer, J. (2022). Leigh-Like Syndrome With a Novel, Complex Phenotype Due to m.10191T>C in Mt-ND3. Cureus, 14(9), e28986. https://doi.org/10.7759/cureus.28986

40. Kori, A., Hori, I., Tanaka, T., Aoyama, K., Ito, K., Hattori, A., Ban, K., Okazaki, Y., Murayama, K., & Saitoh, S. (2019). Transition from Leigh syndrome to MELAS syndrome in a patient with heteroplasmic MT-ND3 m.10158T>C. Brain & development, 41(9), 803–807. https://doi.org/10.1016/j.braindev.2019.05.006

41. Fu, X. L., Zhou, X. X., Shi, Z., & Zheng, W. C. (2019). Adult-onset mitochondrial encephalopathy in association with the MT-ND3 T10158C mutation exhibits unique characteristics: A

case report. World journal of clinical cases, 7(9), 1066–1072. https://doi.org/10.12998/wjcc.v7.i9.1066

42. Newstead, S. M., & Finsterer, J. (2023). Metabolic inflexibility and unusual catabolism in Leigh-like syndrome due to m.10191T>C. Clinical nutrition ESPEN, 56, 149–151. https://doi.org/10.1016/j.clnesp.2023.05.007

43. Kouga, T., Takagi, M., Miyauchi, A., Shimbo, H., Iai, M., Yamashita, S., Murayama, K., Klein, M. B., Miller, G., Goto, T., & Osaka, H. (2018). Japanese Leigh syndrome case treated with EPI-743. Brain & development, 40(2), 145–149. https://doi.org/10.1016/j.braindev.2017.08.005

44. Watson-Fargie, T., Marshall, V., Fullerton, N. E., Leach, V., Pilz, D., Hemingbrough, C. V. Y., Hopton, S., Taylor, R. W., Ng, Y. S., Schaefer, A., Gorman, G. S., & Farrugia, M. E. (2024). Leigh syndrome: an adult presentation of a paediatric disease. Practical neurology, 24(1), 45–50. https://doi.org/10.1136/pn-2023-003862

45. Grosso, S., Carluccio, M. A., Cardaioli, E., Cerase, A., Malandrini, A., Romano, C., Federico, A., & Dotti, M. T. (2017). Complex I deficiency related to T10158C mutation ND3 gene: A further definition of the clinical spectrum. Brain & development, 39(3), 261–265. https://doi.org/10.1016/j.braindev.2016.09.013

46. Mezuki, S., Fukuda, K., Matsushita, T., Fukushima, Y., Matsuo, R., Goto, Y. I., Yasukawa, T., Uchiumi, T., Kang, D., Kitazono, T., & Ago, T. (2017). Isolated and repeated stroke-like episodes in a middle-aged man with a mitochondrial ND3 T10158C mutation: a case report. BMC neurology, 17(1), 217. https://doi.org/10.1186/s12883-017-1001-4

47. Mukai, M., Nagata, E., Mizuma, A., Yamano, M., Sugaya, K., Nishino, I., Goto, Y. I., & Takizawa, S. (2017). Adult-onset Mitochondrial Myopathy, Encephalopathy, Lactic Acidosis, and Stroke (MELAS)-like Encephalopathy Diagnosed Based on the Complete Sequencing of Mitochondrial DNA Extracted from Biopsied Muscle without any Myopathic Changes. Internal medicine (Tokyo, Japan), 56(1), 95–99. https://doi.org/10.2169/internalmedicine.56.7301

48. Tolomeo, D., Rubegni, A., Severino, M., Pochiero, F., Bruno, C., Cassandrini, D., Madeo, A., Doccini, S., Pedemonte, M., Rossi, A., D'Amore, F., Donati, M. A., Di Rocco, M., Santorelli, F. M., & Nesti, C. (2019). Clinical and neuroimaging features of the m.10197G>A mtDNA mutation: New case reports and expansion of the phenotype variability. Journal of the neurological sciences, 399, 69–75. https://doi.org/10.1016/j.jns.2019.02.010

49. Calvo, S. E., Tucker, E. J., Compton, A. G., Kirby, D. M., Crawford, G., Burtt, N. P., Rivas, M., Guiducci, C., Bruno, D. L., Goldberger, O. A., Redman, M. C., Wiltshire, E., Wilson, C. J., Altshuler, D., Gabriel, S. B., Daly, M. J., Thorburn, D. R., & Mootha, V. K. (2010). High-throughput, pooled sequencing identifies mutations in NUBPL and FOXRED1 in human complex I deficiency. Nature genetics, 42(10), 851–858. https://doi.org/10.1038/ng.659

50. Fassone, E., Duncan, A. J., Taanman, J. W., Pagnamenta, A. T., Sadowski, M. I., Holand, T., Qasim, W., Rutland, P., Calvo, S. E., Mootha, V. K., Bitner-Glindzicz, M., & Rahman, S. (2010). FOXRED1, encoding an FAD-dependent oxidoreductase complex-I-specific molecular chaperone, is mutated in infantile-onset mitochondrial encephalopathy. Human molecular genetics, 19(24), 4837–4847. https://doi.org/10.1093/hmg/ddq414

51. Gouiza, I., Hechmi, M., Zioudi, A., Dallali, H., Kheriji, N., Charif, M., Le Mao, M., Galai, S., Kraoua, L., Ben Youssef-Turki, I., Kraoua, I., Lenaers, G., & Kefi, R. (2024). Expanding the genetic spectrum of mitochondrial diseases in Tunisia: novel variants revealed by whole-exome sequencing. Frontiers in genetics, 14, 1259826. https://doi.org/10.3389/fgene.2023.1259826

52. Hu, C., Xu, Q., Shen, J., & Wang, Y. (2021). Clinical and Genetic Characteristics of Mitochondrial Encephalopathy Due to FOXRED1 Mutations: Two Chinese Case Reports and a Review of the Literature. Frontiers in neurology, 12, 633397. https://doi.org/10.3389/fneur.2021.633397

53. Barbosa-Gouveia, S., González-Vioque, E., Borges, F., Gutiérrez-Solana, L., Wintjes, L., Kappen, A., van den Heuvel, L., Leis, R., Rodenburg, R., & Couce, M. L. (2019). Identification and Characterization of New Variants in FOXRED1 Gene Expands the Clinical Spectrum Associated with Mitochondrial Complex I Deficiency. Journal of clinical medicine, 8(8), 1262. https://doi.org/10.3390/jcm8081262

54. Apatean, D., Rakic, B., Brunel-Guitton, C., Hendson, G., Bai, R., Sargent, M. A., Lavoie, P. M., Patel, M., & Stockler-Ipsiroglu, S. (2019). Congenital lactic acidosis, cerebral cysts and pulmonary hypertension in an infant with FOXRED1 related complex I deficiency. Molecular genetics and metabolism reports, 18, 32–38. https://doi.org/10.1016/j.ymgmr.2018.12.006

59. Haack, T. B., Danhauser, K., Haberberger, B., Hoser, J., Strecker, V., Boehm, D., Uziel, G., Lamantea, E., Invernizzi, F., Poulton, J., Rolinski, B., Iuso, A., Biskup, S., Schmidt, T., Mewes, H. W., Wittig, I., Meitinger, T., Zeviani, M., & Prokisch, H. (2010). Exome sequencing identifies ACAD9 mutations as a cause of complex I deficiency. Nature genetics, 42(12), 1131–1134. https://doi.org/10.1038/ng.706

60. He, M., Rutledge, S. L., Kelly, D. R., Palmer, C. A., Murdoch, G., Majumder, N., Nicholls, R. D., Pei, Z., Watkins, P. A., & Vockley, J. (2007). A new genetic disorder in mitochondrial fatty acid beta-oxidation: ACAD9 deficiency. American journal of human genetics, 81(1), 87–103. https://doi.org/10.1086/519219

61. Dewulf, J. P., Barrea, C., Vincent, M. F., De Laet, C., Van Coster, R., Seneca, S., Marie, S., & Nassogne, M. C. (2016). Evidence of a wide spectrum of cardiac involvement due to ACAD9 mutations: Report on nine patients. Molecular genetics and metabolism, 118(3), 185–189. https://doi.org/10.1016/j.ymgme.2016.05.005

62. Lagoutte-Renosi, J., Ségalas-Milazzo, I., Crahes, M., Renosi, F., Menu-Bouaouiche, L., Torre, S., Lardennois, C., Rio, M., Marret, S., Brasse-Lagnel, C., Laquerrière, A., & Bekri, S. (2015). Lethal Neonatal Progression of Fetal Cardiomegaly Associated to ACAD9 Deficiency. JIMD reports, 28, 1–10. Advance online publication. https://doi.org/10.1007/8904_2015_499

63. Aintablian, H. K., Narayanan, V., Belnap, N., Ramsey, K., & Grebe, T. A. (2016). An atypical presentation of ACAD9 deficiency: Diagnosis by whole exome sequencing broadens the phenotypic spectrum and alters treatment approach. Molecular genetics and metabolism reports, 10, 38–44. https://doi.org/10.1016/j.ymgmr.2016.12.005

64. Monda, E., Lioncino, M., Caiazza, M., Simonelli, V., Nesti, C., Rubino, M., Perna, A., Mauriello, A., Budillon, A., Pota, V., Bruno, G., Varone, A., Nigro, V., Santorelli, F. M., Pacileo, G., Russo, M. G., Frisso, G., Sampaolo, S., & Limongelli, G. (2023). Clinical, Genetic, and Histological Characterization of Patients with Rare Neuromuscular and Mitochondrial Diseases Presenting with Different Cardiomyopathy Phenotypes. International journal of molecular sciences, 24(10), 9108. https://doi.org/10.3390/ijms24109108

65. Jacobi-Polishook, T., Yosha-Orpaz, N., Sagi, Y., Lev, D., & Lerman-Sagie, T. (2020). Successful pregnancy in a patient with mitochondrial cardiomyopathy due to ACAD9 deficiency. JIMD reports, 56(1), 9–13. https://doi.org/10.1002/jmd2.12157

66. Garone, C., Donati, M. A., Sacchini, M., Garcia-Diaz, B., Bruno, C., Calvo, S., Mootha, V. K., & Dimauro, S. (2013). Mitochondrial encephalomyopathy due to a novel mutation in ACAD9. JAMA neurology, 70(9), 1177–1179. https://doi.org/10.1001/jamaneurol.2013.3197

67. Schrank, B., Schoser, B., Klopstock, T., Schneiderat, P., Horvath, R., Abicht, A., Holinski-Feder, E., & Augustis, S. (2017). Lifetime exercise intolerance with lactic acidosis as key manifestation of novel compound heterozygous ACAD9 mutations causing complex I deficiency. Neuromuscular disorders : NMD, 27(5), 473–476. https://doi.org/10.1016/j.nmd.2017.02.005

68. Collet, M., Assouline, Z., Bonnet, D., Rio, M., Iserin, F., Sidi, D., Goldenberg, A., Lardennois, C., Metodiev, M. D., Haberberger, B., Haack, T., Munnich, A., Prokisch, H., & Rötig, A. (2016). High incidence and variable clinical outcome of cardiac hypertrophy due to ACAD9 mutations in childhood. European journal of human genetics : EJHG, 24(8), 1112–1116. https://doi.org/10.1038/ejhg.2015.264

69. Lek, M., Karczewski, K. J., Minikel, E. V., Samocha, K. E., Banks, E., Fennell, T., O'Donnell-Luria, A. H., Ware, J. S., Hill, A. J., Cummings, B. B., Tukiainen, T., Birnbaum, D. P., Kosmicki, J. A., Duncan, L. E., Estrada, K., Zhao, F., Zou, J., Pierce-Hoffman, E., Berghout, J., Cooper, D. N., … Exome Aggregation Consortium (2016). Analysis of protein-coding genetic variation in 60,706 humans. Nature, 536(7616), 285–291. https://doi.org/10.1038/nature19057

70. Scholte, H. R., Busch, H. F., Bakker, H. D., Bogaard, J. M., Luyt-Houwen, I. E., & Kuyt, L. P. (1995). Riboflavin-responsive complex I deficiency. Biochimica et biophysica acta, 1271(1), 75–83. https://doi.org/10.1016/0925-4439(95)00013-t

71. Kohda, M., Tokuzawa, Y., Kishita, Y., Nyuzuki, H., Moriyama, Y., Mizuno, Y., Hirata, T., Yatsuka, Y., Yamashita-Sugahara, Y., Nakachi, Y., Kato, H., Okuda, A., Tamaru, S., Borna, N. N., Banshoya, K., Aigaki, T., Sato-Miyata, Y., Ohnuma, K., Suzuki, T., Nagao, A., … Okazaki, Y. (2016). A Comprehensive Genomic Analysis Reveals the Genetic Landscape of Mitochondrial Respiratory Chain Complex Deficiencies. PLoS genetics, 12(1), e1005679. https://doi.org/10.1371/journal.pgen.1005679

72. Leslie, N., Wang, X., Peng, Y., Valencia, C. A., Khuchua, Z., Hata, J., Witte, D., Huang, T., & Bove, K. E. (2016). Neonatal multiorgan failure due to ACAD9 mutation and complex I deficiency with mitochondrial hyperplasia in liver, cardiac myocytes, skeletal muscle, and renal tubules. Human pathology, 49, 27–32. https://doi.org/10.1016/j.humpath.2015.09.039

73. Distelmaier, F., Haack, T. B., Wortmann, S. B., Mayr, J. A., & Prokisch, H. (2017). Treatable mitochondrial diseases: cofactor metabolism and beyond. Brain : a journal of neurology, 140(2), e11. https://doi.org/10.1093/brain/aww303

74. Repp, B. M., Mastantuono, E., Alston, C. L., Schiff, M., Haack, T. B., Rötig, A., Ardissone, A., Lombès, A., Catarino, C. B., Diodato, D., Schottmann, G., Poulton, J., Burlina, A., Jonckheere, A., Munnich, A., Rolinski, B., Ghezzi, D., Rokicki, D., Wellesley, D., Martinelli, D., … Wortmann, S. (2018). Clinical, biochemical and genetic spectrum of 70 patients with ACAD9 deficiency: is riboflavin supplementation effective?. Orphanet journal of rare diseases, 13(1), 120. https://doi.org/10.1186/s13023-018-0784-8

75. Blickhäuser, B., Stenton, S. L., Neuhofer, C. M., Floride, E., Nesbitt, V., Fratter, C., Koch, J., Kauffmann, B., Catarino, C., Schlieben, L. D., Kopajtich, R., Carelli, V., Sadun, A. A., McFarland, R., Fang, F., La Morgia, C., Paquay, S., Nassogne, M. C., Ghezzi, D., Lamperti, C., … Prokisch, H. (2024). Digenic Leigh syndrome on the background of the m.11778G>A Leber hereditary optic neuropathy variant. Brain : a journal of neurology, 147(6), 1967–1974. https://doi.org/10.1093/brain/awae057

76. Vacchiano, V., Caporali, L., La Morgia, C., Carbonelli, M., Amore, G., Bartolomei, I., Cascavilla, M. L., Barboni, P., Lamperti, C., Catania, A., Chan, J. W., Karanja, R., Sadun, A. A., Liguori, R., Bianchi, A., Gavazzi, G., Mascalchi, M., Salvi, F., & Carelli, V. (2021). The m.3890G>A/MT-ND1 mtDNA rare pathogenic variant: Expanding clinical and MRI phenotypes. Mitochondrion, 60, 142–149. https://doi.org/10.1016/j.mito.2021.08.007

77. Xu, M., Kopajtich, R., Elstner, M., Li, H., Liu, Z., Wang, J., Prokisch, H., & Fang, F. (2022). Identification of a novel m.3955G > A variant in MT-ND1 associated with Leigh syndrome. Mitochondrion, 62, 13–23. https://doi.org/10.1016/j.mito.2021.10.002

78. Spangenberg, L., Graña, M., Greif, G., Suarez-Rivero, J. M., Krysztal, K., Tapié, A., Boidi, M., Fraga, V., Lemes, A., Gueçaimburú, R., Cerisola, A., Sánchez-Alcázar, J. A., Robello, C., Raggio, V., & Naya, H. (2016). 3697G>A in MT-ND1 is a causative mutation in mitochondrial disease. Mitochondrion, 28, 54–59. https://doi.org/10.1016/j.mito.2016.03.006

79. Martínez-Romero, Í., Herrero-Martín, M. D., Llobet, L., Emperador, S., Martín-Navarro, A., Narberhaus, B., Ascaso, F. J., López-Gallardo, E., Montoya, J., & Ruiz-Pesini, E. (2014). New MT-ND1 pathologic mutation for Leber hereditary optic neuropathy. Clinical & experimental ophthalmology, 42(9), 856–864. https://doi.org/10.1111/ceo.12355

80. Rákosníková, T., Kelifová, S., Štufková, H., Lokvencová, K., Lišková, P., Kousal, B., Honzík, T., Hansíková, H., Martínek, V., & Tesařová, M. (2023). Case report: A rare variant m.4135T>C in the MT-ND1 gene leads to Leber hereditary optic neuropathy and altered respiratory chain supercomplexes. Frontiers in genetics, 14, 1182288. https://doi.org/10.3389/fgene.2023.1182288

81. Hayashi, Y., Iwasaki, Y., Yoshikura, N., Yamada, M., Kimura, A., Inuzuka, T., Miyahara, H., Goto, Y., Nishino, I., Yoshida, M., & Shimohata, T. (2021). Clinicopathological findings of a mitochondrial encephalopathy, lactic acidosis, and stroke-like episodes/Leigh syndrome overlap patient with a novel m.3482A>G mutation in MT-ND1. Neuropathology : official journal of the Japanese Society of Neuropathology, 41(1), 84–90. https://doi.org/10.1111/neup.12709

82. La Morgia, C., Caporali, L., Gandini, F., Olivieri, A., Toni, F., Nassetti, S., Brunetto, D., Stipa, C., Scaduto, C., Parmeggiani, A., Tonon, C., Lodi, R., Torroni, A., & Carelli, V. (2014). Association of the mtDNA m.4171C>A/MT-ND1 mutation with both optic neuropathy and bilateral brainstem lesions. BMC neurology, 14, 116. https://doi.org/10.1186/1471-2377-14-116

83. Kirby, D. M., McFarland, R., Ohtake, A., Dunning, C., Ryan, M. T., Wilson, C., Ketteridge, D., Turnbull, D. M., Thorburn, D. R., & Taylor, R. W. (2004). Mutations of the mitochondrial ND1 gene as a cause of MELAS. Journal of medical genetics, 41(10), 784–789. https://doi.org/10.1136/jmg.2004.020537

84. Carreño-Gago, L., Gamez, J., Cámara, Y., Alvarez de la Campa, E., Aller-Alvarez, J. S., Moncho, D., Salvado, M., Galan, A., de la Cruz, X., Pinós, T., & García-Arumí, E. (2017). Identification and characterization of the novel point mutation m.3634A>G in the mitochondrial MT-ND1 gene associated with LHON syndrome. Biochimica et biophysica acta. Molecular basis of disease, 1863(1), 182–187. https://doi.org/10.1016/j.bbadis.2016.09.002

85. Wray, C. D., Friederich, M. W., du Sart, D., Pantaleo, S., Smet, J., Kucera, C., Fenton, L., Scharer, G., Van Coster, R., & Van Hove, J. L. (2013). A new mutation in MT-ND1 m.3928G>C p.V208L causes Leigh disease with infantile spasms. Mitochondrion, 13(6), 656–661. https://doi.org/10.1016/j.mito.2013.09.004

86. Lin, J., Zhao, C. B., Lu, J. H., Wang, H. J., Zhu, W. H., Xi, J. Y., Lu, J., Luo, S. S., Ma, D., Wang, Y., Xiao, B. G., & Lu, C. Z. (2014). Novel mutations m.3959G>A and m.3995A>G in

mitochondrial gene MT-ND1 associated with MELAS. Mitochondrial DNA, 25(1), 56–62. https://doi.org/10.3109/19401736.2013.779259

87. Ammar, M., Tabebi, M., Sfaihi, L., Alila-Fersi, O., Maalej, M., Felhi, R., Chabchoub, I., Keskes, L., Hachicha, M., Fakhfakh, F., & Mkaouar-Rebai, E. (2016). Mutational screening in patients with profound sensorineural hearing loss and neurodevelopmental delay: Description of a novel m.3861A > C mitochondrial mutation in the MT-ND1 gene. Biochemical and biophysical research communications, 474(4), 702–708. https://doi.org/10.1016/j.bbrc.2016.05.014

88. Delmiro, A., Rivera, H., García-Silva, M. T., García-Consuegra, I., Martín-Hernández, E., Quijada-Fraile, P., de Las Heras, R. S., Moreno-Izquierdo, A., Martín, M. Á., Arenas, J., & Martínez-Azorín, F. (2013). Whole-exome sequencing identifies a variant of the mitochondrial MT-ND1 gene associated with epileptic encephalopathy: west syndrome evolving to Lennox-Gastaut syndrome. Human mutation, 34(12), 1623–1627. https://doi.org/10.1002/humu.22445

89. Petrovic Pajic, S., Fakin, A., Sustar Habjan, M., Jarc-Vidmar, M., & Hawlina, M. (2023). Leber Hereditary Optic Neuropathy (LHON) in Patients with Presumed Childhood Monocular Amblyopia. Journal of clinical medicine, 12(20), 6669. https://doi.org/10.3390/jcm12206669

90. Mkaouar-Rebai, E., Ammar, M., Sfaihi, L., Alila-Fersi, O., Maalej, M., Felhi, R., Hachicha, M., & Fakhfakh, F. (2021). Mitochondrial disease patients with novel ND4 12058A > C and ND1 m.3911A > G variations: implications for a role in the phenotype following a bioinformatic investigation. Molecular biology reports, 48(5), 4373–4382. https://doi.org/10.1007/s11033-021-06452-4

91. Negishi, Y., Hattori, A., Takeshita, E., Sakai, C., Ando, N., Ito, T., Goto, Y., & Saitoh, S. (2014). Homoplasmy of a mitochondrial 3697G>A mutation causes Leigh syndrome. Journal of human genetics, 59(7), 405–407. https://doi.org/10.1038/jhg.2014.41

92. Zifa, E., Theotokis, P., Kaminari, A., Maridaki, H., Leze, H., Petsiava, E., Mamuris, Z., & Stathopoulos, C. (2008). A novel G3337A mitochondrial ND1 mutation related to cardiomyopathy co-segregates with tRNALeu(CUN) A12308G and tRNAThr C15946T mutations. Mitochondrion, 8(3), 229–236. https://doi.org/10.1016/j.mito.2008.04.001

93. Murray, J. J., Nolan, K. W., McClelland, C., & Lee, M. S. (2017). Leber Hereditary Optic Neuropathy: Visual Recovery in a Patient With the Rare m.3890G>A Point Mutation. Journal of neuro-ophthalmology : the official journal of the North American Neuro-Ophthalmology Society, 37(2), 166–171. https://doi.org/10.1097/WNO.0000000000000462

94. Blakely, E. L., de Silva, R., King, A., Schwarzer, V., Harrower, T., Dawidek, G., Turnbull, D. M., & Taylor, R. W. (2005). LHON/MELAS overlap syndrome associated with a mitochondrial MTND1 gene mutation. European journal of human genetics : EJHG, 13(5), 623–627. https://doi.org/10.1038/sj.ejhg.5201363

95. Blakely, E. L., Rennie, K. J., Jones, L., Elstner, M., Chrzanowska-Lightowlers, Z. M., White, C. B., Shield, J. P., Pilz, D. T., Turnbull, D. M., Poulton, J., & Taylor, R. W. (2006). Sporadic intragenic inversion of the mitochondrial DNA MTND1 gene causing fatal infantile lactic acidosis. Pediatric research, 59(3), 440–444. https://doi.org/10.1203/01.pdr.0000198771.78290.c4

96. Gorman, G. S., Blakely, E. L., Hornig-Do, H. T., Tuppen, H. A., Greaves, L. C., He, L., Baker, A., Falkous, G., Newman, J., Trenell, M. I., Lecky, B., Petty, R. K., Turnbull, D. M., McFarland, R., & Taylor, R. W. (2015). Novel MTND1 mutations cause isolated exercise intolerance, complex I deficiency and increased assembly factor expression. Clinical science (London, England : 1979), 128(12), 895–904. https://doi.org/10.1042/CS20140705

97. Blickhäuser, B., Stenton, S. L., Neuhofer, C. M., Floride, E., Nesbitt, V., Fratter, C., Koch, J., Kauffmann, B., Catarino, C., Schlieben, L. D., Kopajtich, R., Carelli, V., Sadun, A. A., McFarland, R., Fang, F., La Morgia, C., Paquay, S., Nassogne, M. C., Ghezzi, D., Lamperti, C., … Prokisch, H. (2024). Digenic Leigh syndrome on the background of the m.11778G>A Leber hereditary optic neuropathy variant. Brain : a journal of neurology, 147(6), 1967–1974. https://doi.org/10.1093/brain/awae057

98. Liutkeviciene, R., Sidaraite, A., Kuliaviene, L., Glebauskiene, B., Jurkute, N., Aluzaite-Baranauskiene, L., Gelzinis, A., & Zemaitiene, R. (2021). A Typical Case Presentation with Spontaneous Visual Recovery in Patient Diagnosed with Leber Hereditary Optic Neuropathy due to Rare Point Mutation in MT-ND4 Gene (m.11253T>C) and Literature Review. Medicina (Kaunas, Lithuania), 57(3), 202. https://doi.org/10.3390/medicina57030202

99. Berardo, A., Emmanuele, V., Vargas, W., Tanji, K., Naini, A., & Hirano, M. (2020). Leber hereditary optic neuropathy plus dystonia, and transverse myelitis due to double mutations in MT-ND4 and MT-ND6. Journal of neurology, 267(3), 823–829. https://doi.org/10.1007/s00415-019-09619-z

100. Endres, D., Süß, P., Maier, S. J., Friedel, E., Nickel, K., Ziegler, C., Fiebich, B. L., Glocker, F. X., Stock, F., Egger, K., Lange, T., Dacko, M., Venhoff, N., Erny, D., Doostkam, S., Komlosi, K., Domschke, K., & Tebartz van Elst, L. (2019). New Variant of MELAS Syndrome With Executive Dysfunction, Heteroplasmic Point Mutation in the MT-ND4 Gene (m.12015T>C; p.Leu419Pro) and Comorbid Polyglandular Autoimmune Syndrome Type 2. Frontiers in immunology, 10, 412. https://doi.org/10.3389/fimmu.2019.00412

101. Mkaouar-Rebai, E., Ammar, M., Sfaihi, L., Alila-Fersi, O., Maalej, M., Felhi, R., Hachicha, M., & Fakhfakh, F. (2021). Mitochondrial disease patients with novel ND4 12058A > C and ND1 m.3911A > G variations: implications for a role in the phenotype following a bioinformatic investigation. Molecular biology reports, 48(5), 4373–4382. https://doi.org/10.1007/s11033-021-06452-4

102. Aung, M. H., Volpe, N. J., Choi, D. J., Stein, J. M., Goldstein, A., & Liu, G. T. (2022). A Tale of Progressive Painless Vision Loss in a 64-Year-Old Man Due to Leber Hereditary Optic Neuropathy. Journal of neuro-ophthalmology : the official journal of the North American Neuro-Ophthalmology Society, 42(3), 390–395. https://doi.org/10.1097/WNO.0000000000001651

103. Perez Giraldo, G. S., Graham, E. L., VanHaerents, S., & Balabanov, R. (2023). Case report: Use of granulocyte-colony stimulating factor as an immunomodulatory therapy in a patient with neuromyelitis optica spectrum disorder and comorbid immunodeficiency. Frontiers in neurology, 14, 1240356. https://doi.org/10.3389/fneur.2023.1240356

104. Lnu, P., Sehgal, V., Bhalla Sehgal, L., Gulati, N., & Kapila, S. (2022). A Case of a 23-Year-Old Male With Leber Hereditary Optic Neuropathy With a Rare Mutation. Cureus, 14(10), e30198. https://doi.org/10.7759/cureus.30198

105. Zhang, A. M., Jia, X., Guo, X., Zhang, Q., & Yao, Y. G. (2012). Mitochondrial DNA mutation m.10680G > A is associated with Leber hereditary optic neuropathy in Chinese patients. Journal of translational medicine, 10, 43. https://doi.org/10.1186/1479-5876-10-43

106. Wilkins, S. R., Yu, A. W., Steigerwald, C., Tanji, K., Iglesias, A. D., Hirano, M., Kister, I., Riley, C. S., & Abreu, N. J. (2023). Two cases of MT-ND5-related mitochondrial disorder misdiagnosed as seronegative neuromyelitis optica spectrum disorder. Multiple sclerosis (Houndmills, Basingstoke, England), 29(7), 892–897. https://doi.org/10.1177/13524585231172947

107. Barone, V., La Morgia, C., Caporali, L., Fiorini, C., Carbonelli, M., Gramegna, L. L., Bartiromo, F., Tonon, C., Morandi, L., Liguori, R., Petrini, A., Brugnano, R., Del Sordo, R., Covarelli, C., Morroni, M., Lodi, R., & Carelli, V. (2022). Case Report: Optic Atrophy and Nephropathy With m.13513G>A/MT-ND5 mtDNA Pathogenic Variant. Frontiers in genetics, 13, 887696. https://doi.org/10.3389/fgene.2022.887696

108. Ghosh, R., Dubey, S., Bhuin, S., Lahiri, D., Ray, B. K., & Finsterer, J. (2022). MELAS with multiple stroke-like episodes due to the variant m.13513G>A in MT-ND5. Clinical case reports, 10(2), e05361. https://doi.org/10.1002/ccr3.5361

109. Yahata, N., Matsumoto, Y., Omi, M., Yamamoto, N., & Hata, R. (2017). TALEN-mediated shift of mitochondrial DNA heteroplasmy in MELAS-iPSCs with m.13513G>A mutation. Scientific reports, 7(1), 15557. https://doi.org/10.1038/s41598-017-15871-y

110. Omata, F., Nakazawa, H., Nakano, M., & Arimori, S. (1990). Erythrocyte superoxide dismutase in various hematological diseases. The Tokai journal of experimental and clinical medicine, 15(2-3), 99–106.

111. Vázquez-Justes, D., Carreño-Gago, L., García-Arumi, E., Traveset, A., Montoya, J., Ruiz-Pesini, E., López, R., & Brieva, L. (2019). Mitochondrial m.13513G>A Point Mutation in ND5 in a 16-Year-Old Man with Leber Hereditary Optic Neuropathy Detected by Next-Generation Sequencing. Journal of pediatric genetics, 8(4), 231–234. https://doi.org/10.1055/s-0039-1691812

112. Wang, Z., Qi, X. K., Yao, S., Chen, B., Luan, X., Zhang, W., Han, M., & Yuan, Y. (2010). Phenotypic patterns of MELAS/LS overlap syndrome associated with m.13513G>A mutation, and neuropathological findings in one autopsy case. Neuropathology : official journal of the Japanese Society of Neuropathology, 30(6), 606–614. https://doi.org/10.1111/j.1440-1789.2010.01115.x

113. Finsterer, J., & Hayman, J. (2022). Mitochondrial Encephalopathy, Lactic Acidosis and Stroke-Like Episodes/Leigh Overlap Syndrome Due to Variant m.13513G>A in MT-ND5. Cureus, 14(5), e24746. https://doi.org/10.7759/cureus.24746

114. Engvall, M., Kawasaki, A., Carelli, V., Wibom, R., Bruhn, H., Lesko, N., Schober, F. A., Wredenberg, A., Wedell, A., & Träisk, F. (2021). Case Report: A Novel Mutation in the Mitochondrial MT-ND5 Gene Is Associated With Leber Hereditary Optic Neuropathy (LHON). Frontiers in neurology, 12, 652590. https://doi.org/10.3389/fneur.2021.652590

115. Zhou, N., Tang, L., Jiang, Y., Qin, S., Cui, J., Wang, Y., Zhu, W., Zhao, W., Pan, C., & Shu, X. (2019). Whole-exome sequencing reveals a novel mutation of MT-ND5 gene in a mitochondrial cardiomyopathy pedigree: Patients who show biventricular hypertrophy, hyperlactacidemia, pulmonary hypertension, and decreased exercise tolerance. Anatolian journal of cardiology, 21(1), 18–24. https://doi.org/10.14744/AnatolJCardiol.2018.53258

116. Ayalon, N., Flore, L. A., Christensen, T. G., & Sam, F. (2013). Mitochondrial encoded NADH dehydrogenase 5 (MT-ND5) gene point mutation presents as late onset cardiomyopathy. International journal of cardiology, 167(5), e143–e145. https://doi.org/10.1016/j.ijcard.2013.04.018

117. Sonam, K., Bindu, P. S., Taly, A. B., Govindaraju, C., Gayathri, N., Arvinda, H. R., Nagappa, M., Sinha, S., Khan, N. A., Govindaraj, P., & Thangaraj, K. (2015). Clinical and Neuroimaging Features in Two Children with Mutations in the Mitochondrial ND5 Gene. Neuropediatrics, 46(4), 277–281. https://doi.org/10.1055/s-0035-1550149

118. McKenzie, M., Liolitsa, D., Akinshina, N., Campanella, M., Sisodiya, S., Hargreaves, I., Nirmalananthan, N., Sweeney, M. G., Abou-Sleiman, P. M., Wood, N. W., Hanna, M. G., & Duchen, M. R. (2007). Mitochondrial ND5 gene variation associated with encephalomyopathy and

mitochondrial ATP consumption. The Journal of biological chemistry, 282(51), 36845–36852. https://doi.org/10.1074/jbc.M704158200

119. Naini, A. B., Lu, J., Kaufmann, P., Bernstein, R. A., Mancuso, M., Bonilla, E., Hirano, M., & DiMauro, S. (2005). Novel mitochondrial DNA ND5 mutation in a patient with clinical features of MELAS and MERRF. Archives of neurology, 62(3), 473–476. https://doi.org/10.1001/archneur.62.3.473

120. Downham, E., Winterthun, S., Nakkestad, H. L., Hirth, A., Halvorsen, T., Taylor, R. W., & Bindoff, L. A. (2008). A novel mitochondrial ND5 (MTND5) gene mutation giving isolated exercise intolerance. Neuromuscular disorders : NMD, 18(4), 310–314. https://doi.org/10.1016/j.nmd.2008.01.003

121. Fang, H., Shi, H., Li, X., Sun, D., Li, F., Li, B., Ding, Y., Ma, Y., Liu, Y., Zhang, Y., Shen, L., Bai, Y., Yang, Y., & Lu, J. (2015). Exercise intolerance and developmental delay associated with a novel mitochondrial ND5 mutation. Scientific reports, 5, 10480. https://doi.org/10.1038/srep10480

122. Blok, M. J., Spruijt, L., de Coo, I. F., Schoonderwoerd, K., Hendrickx, A., & Smeets, H. J. (2007). Mutations in the ND5 subunit of complex I of the mitochondrial DNA are a frequent cause of oxidative phosphorylation disease. Journal of medical genetics, 44(4), e74. https://doi.org/10.1136/jmg.2006.045716

123. Kolarova, H., Liskova, P., Tesarova, M., Kucerova Vidrova, V., Forgac, M., Zamecnik, J., Hansikova, H., & Honzik, T. (2016). Unique presentation of LHON/MELAS overlap syndrome caused by m.13046T>C in MTND5. Ophthalmic genetics, 37(4), 419–423. https://doi.org/10.3109/13816810.2015.1092045

124. Slawek, J., Kierdaszuk, B., Tonska, K., Kodron, A., Schinwelski, M., Sitek, E. J., Bartnik, E., Kaminska, A., & Kwiecinski, H. (2012). Mitochondrial encephalopathy in a patient with a 13042G>A de novo mutation. Journal of clinical pathology, 65(12), 1147–1149. https://doi.org/10.1136/jclinpath-2012-200778

125. Kishita, Y., Ishikawa, K., Nakada, K., Hayashi, J. I., Fushimi, T., Shimura, M., Kohda, M., Ohtake, A., Murayama, K., & Okazaki, Y. (2021). A high mutation load of m.14597A>G in MT-ND6 causes Leigh syndrome. Scientific reports, 11(1), 11123. https://doi.org/10.1038/s41598-021-90196-5

126. Rizk, M., Dunya, I., Azar, G., Seif, R., Megarbane, A., & Sadaka, A. (2020). Novel Mutations of mtDNA m.14568G>A/m.14568C>T in MT-ND6 and m.7299A>G in MT-CO1: Evidence of Pathogenicity in Leber Hereditary Optic Neuropathy. Journal of neuro-ophthalmology : the official journal of the North American Neuro-Ophthalmology Society, 40(4), 566–568. https://doi.org/10.1097/WNO.0000000000000973

127. To, L. K., Shah, P. R., Scanga, H. L., Franks, A. L., Cladis, F. P., & Nischal, K. K. (2019). Personalized pediatric ophthalmology: a case report. Journal of AAPOS : the official publication of the American Association for Pediatric Ophthalmology and Strabismus, 23(4), 234–236. https://doi.org/10.1016/j.jaapos.2019.03.003

128. Tarnopolsky, M., Meaney, B., Robinson, B., Sheldon, K., & Boles, R. G. (2013). Severe infantile leigh syndrome associated with a rare mitochondrial ND6 mutation, m.14487T>C. American journal of medical genetics. Part A, 161A(8), 2020–2023. https://doi.org/10.1002/ajmg.a.36000

129. Gunawardena, K., Dissanayake, V. H. W., & Chang, T. (2023). The first genetically authenticated case of Leber hereditary optic neuropathy in Sri Lanka: a case report and review of the literature. Journal of medical case reports, 17(1), 34. https://doi.org/10.1186/s13256-023-03763-x

130. Petrovic Pajic, S., Fakin, A., Sustar Habjan, M., Jarc-Vidmar, M., & Hawlina, M. (2023). Leber Hereditary Optic Neuropathy (LHON) in Patients with Presumed Childhood Monocular Amblyopia. Journal of clinical medicine, 12(20), 6669. https://doi.org/10.3390/jcm12206669

131. Maruo, Y., Ueda, Y., Murayama, K., & Takeda, A. (2021). A case report of Leigh syndrome diagnosed by endomyocardial biopsy. European heart journal. Case reports, 5(2), ytaa582. https://doi.org/10.1093/ehjcr/ytaa582

132. Chen, Y., & Kellom, E. R. (2023). A rare, likely pathogenic variant causing Leber's hereditary optic neuropathy in three-generation females of an African-American family. American journal of ophthalmology case reports, 32, 101936. https://doi.org/10.1016/j.ajoc.2023.101936

133. Sallevelt, S. C., Dreesen, J. C., Drüsedau, M., Hellebrekers, D. M., Paulussen, A. D., Coonen, E., van Golde, R. J., Geraedts, J. P., Gianaroli, L., Magli, M. C., Zeviani, M., Smeets, H. J., & de Die-Smulders, C. E. (2017). PGD for the m.14487 T>C mitochondrial DNA mutation resulted in the birth of a healthy boy. Human reproduction (Oxford, England), 32(3), 698–703. https://doi.org/10.1093/humrep/dew356

134. Leshinsky-Silver, E., Shuvalov, R., Inbar, S., Cohen, S., Lev, D., & Lerman-Sagie, T. (2011). Juvenile Leigh syndrome, optic atrophy, ataxia, dystonia, and epilepsy due to T14487C mutation in the mtDNA-ND6 gene: a mitochondrial syndrome presenting from birth to adolescence. Journal of child neurology, 26(4), 476–481. https://doi.org/10.1177/0883073810384615

135. Bannwarth, S., Abbassi, M., Valéro, R., Fragaki, K., Dubois, N., Vialettes, B., & Paquis-Flucklinger, V. (2011). A novel unstable mutation in mitochondrial DNA responsible for maternally inherited diabetes and deafness. Diabetes care, 34(12), 2591–2593. https://doi.org/10.2337/dc11-1012

136. Yokota, Y., Hara, M., Akimoto, T., Mizoguchi, T., Goto, Y. I., Nishino, I., Kamei, S., & Nakajima, H. (2020). Late-onset MELAS syndrome with mtDNA 14453G→A mutation masquerading as an acute encephalitis: a case report. BMC neurology, 20(1), 247. https://doi.org/10.1186/s12883-020-01818-w

137. Kim, I. S., Ki, C. S., & Park, K. J. (2010). Pediatric-onset dystonia associated with bilateral striatal necrosis and G14459A mutation in a Korean family: a case report. Journal of Korean medical science, 25(1), 180–184. https://doi.org/10.3346/jkms.2010.25.1.180

138. Pojda-Wilczek, D., Wójcik, J., Kmak, B., & Krawczyński, M. R. (2022). Phenotypic Variation of Autosomal Recessive Leber Hereditary Optic Neuropathy (arLHON) in One Family. Diagnostics (Basel, Switzerland), 12(11), 2701. https://doi.org/10.3390/diagnostics12112701

139. Stenton, S. L., Tesarova, M., Sheremet, N. L., Catarino, C. B., Carelli, V., Ciara, E., Curry, K., Engvall, M., Fleming, L. R., Freisinger, P., Iwanicka-Pronicka, K., Jurkiewicz, E., Klopstock, T., Koenig, M. K., Kolářová, H., Kousal, B., Krylova, T., La Morgia, C., Nosková, L., Piekutowska-Abramczuk, D., … Prokisch, H. (2022). DNAJC30 defect: a frequent cause of recessive Leber hereditary optic neuropathy and Leigh syndrome. Brain : a journal of neurology, 145(5), 1624–1631. https://doi.org/10.1093/brain/awac052

140. Zawadzka, M., Krygier, M., Pawłowicz, M., Wilke, M. V. M. B., Rutkowska, K., Gueguen, N., Desquiret-Dumas, V., Klee, E. W., Schimmenti, L. A., Sławek, J., Procaccio, V., Płoski, R., & Mazurkiewicz-Bełdzińska, M. (2022). Expanding the phenotype of DNAJC30-associated Leigh syndrome. Clinical genetics, 102(5), 438–443. https://doi.org/10.1111/cge.14196

141. Giannoccaro, M. P., Morelli, L., Ricciardiello, F., Donadio, V., Bartiromo, F., Tonon, C., Carbonelli, M., Amore, G., Carelli, V., Liguori, R., & La Morgia, C. (2024). Co-occurrence of glial fibrillary acidic protein astrocytopathy in a patient with Leber's hereditary optic neuropathy due to

DNAJC30 mutations. European journal of neurology, e16344. Advance online publication. https://doi.org/10.1111/ene.16344

142. Roomets, E., & Mauring, L. (2024). Autosomal recessive leber hereditary optic neuropathy in a choroideremia carrier. A case report. European journal of ophthalmology, 11206721241254408. Advance online publication. https://doi.org/10.1177/11206721241254408

143. Petrovic Pajic, S., Jarc-Vidmar, M., Fakin, A., Sustar Habjan, M., Brecelj, J., Volk, M., Maver, A., Peterlin, B., & Hawlina, M. (2022). Case report: Long-term follow-up of two patients with LHON caused by DNAJC30:c.152G>A pathogenic variant-case series. Frontiers in neurology, 13, 1003046. https://doi.org/10.3389/fneur.2022.1003046

144. Nesti, C., Ticci, C., Rubegni, A., Doccini, S., Scaturro, G., Vetro, A., Guerrini, R., Santorelli, F. M., & Procopio, E. (2023). Additive effect of DNAJC30 and NDUFA9 mutations causing Leigh syndrome. Journal of neurology, 270(6), 3266–3269. https://doi.org/10.1007/s00415-023-11673-7

145. Shen, C., Wang, K., Li, W., Serrano, A., Powers, K., Zhang, C., Chen, J., & Sun, M. (2022). A homozygous nonsense mutation in DNAJC30 causes Leber's hereditary optic neuropathy with Leigh-like phenotypes. Genes & diseases, 10(4), 1165–1168. https://doi.org/10.1016/j.gendis.2022.09.011

146. Mauring, L., Puusepp, S., Parik, M., Roomets, E., Teek, R., Reimand, T., Pajusalu, S., Kaljurand, K., & Õunap, K. (2023). Autosomal recessive Leber's hereditary optic neuropathy caused by a homozygous variant in DNAJC30 gene. European journal of medical genetics, 66(9), 104821. https://doi.org/10.1016/j.ejmg.2023.104821

147. Major, T. C., Arany, E. S., Schon, K., Simo, M., Karcagi, V., van den Ameele, J., Yu Wai Man, P., Chinnery, P. F., Olimpio, C., & Horvath, R. (2023). Case report: Mutations in DNAJC30 causing autosomal recessive Leber hereditary optic neuropathy are common amongst Eastern European individuals. Frontiers in neurology, 14, 1292320. https://
